# *Aloe* Genus Plants: From Farm to Food Applications and Phytopharmacotherapy

**DOI:** 10.3390/ijms19092843

**Published:** 2018-09-19

**Authors:** Bahare Salehi, Sevil Albayrak, Hubert Antolak, Dorota Kręgiel, Ewelina Pawlikowska, Mehdi Sharifi-Rad, Yadav Uprety, Patrick Valere Tsouh Fokou, Zubaida Yousef, Zainul Amiruddin Zakaria, Elena Maria Varoni, Farukh Sharopov, Natália Martins, Marcello Iriti, Javad Sharifi-Rad

**Affiliations:** 1 Medical Ethics and Law Research Center, Shahid Beheshti University of Medical Sciences, Tehran 88777539, Iran; bahar.salehi007@gmail.com; 2 Student Research Committee, Shahid Beheshti University of Medical Sciences, Tehran 22439789, Iran; 3 Department of Biology, Science Faculty, Erciyes University, Kayseri 38039, Turkey; salbayrak@erciyes.edu.tr; 4 Institute of Fermentation Technology and Microbiology, Faculty of Biotechnology and Food Science, Lodz University of Technology, Wolczanska 171/173, 90-924 Lodz, Poland; hubert.antolak@gmail.com (H.A.); dorota.kregiel@p.lodz.pl (D.K.); ewelina.pawlikowska@dokt.p.lodz.pl (E.P.); 5 Department of Medical Parasitology, Zabol University of Medical Sciences, Zabol 61663-335, Iran; 6 Research Centre for Applied Science and Technology (RECAST), Tribhuvan University, P.O. Box 1030 Kirtipur, Kathmandu, Nepal; yadavuprety@gmail.com; 7 Antimicrobial and Biocontrol Agents Unit, Department of Biochemistry, Faculty of Science, University of Yaounde 1, Ngoa Ekelle, Annex Fac. Sci, P.O. Box 812 Yaounde, Cameroon; tsouh80@yahoo.fr; 8 Department of Botany, Lahore College for Women University, Jail Road Lahore 54000, Pakistan; mussabuswaeshal@hotmail.com; 9 Department of Biomedical Sciences, Faculty of Medicine and Health Sciences, Universiti Putra Malaysia, Serdang 43400, Malaysia; dr_zaz@yahoo.com; 10 Integrative Pharmacogenomics Institute (iPROMISE), Level 7, FF3 Building, Universiti Teknologi MARA, Puncak Alam 42300, Malaysia; 11 Department of Biomedical, Surgical and Dental Sciences, Milan State University, via Beldiletto 1/3, 20100 Milan, Italy; elena.varoni@unimi.it; 12 National Interuniversity Consortium of Materials Science and Technology, via G. Giusti 9, 50121 Firenze, Italy; 13 Department of Pharmaceutical Technology, Avicenna Tajik State Medical University, Rudaki 139, Dushanbe 734003, Tajikistan; 14 Faculty of Medicine, University of Porto, Alameda Prof. Hernâni Monteiro, 4200-319 Porto, Portugal; 15 Institute for Research and Innovation in Health (i3S), University of Porto, 4200-135 Porto, Portugal; 16 Department of Agricultural and Environmental Sciences, Milan State University, via G. Celoria 2, 20133 Milan, Italy; 17 Phytochemistry Research Center, Shahid Beheshti University of Medical Sciences, Tehran 11369, Iran; 18 Department of Chemistry, Richardson College for the Environmental Science Complex, The University of Winnipeg, 599 Portage Avenue, Winnipeg, MB R3B 2G3, Canada

**Keywords:** *Aloe* species, aloin, plicataloside, isovitexin, aloe emodin, aloesin, aloinoside

## Abstract

*Aloe* genus plants, distributed in Old World, are widely known and have been used for centuries as topical and oral therapeutic agents due to their health, beauty, medicinal, and skin care properties. Among the well-investigated *Aloe* species are *A. arborescens*, *A. barbadensis*, *A. ferox*, and *A. vera*. Today, they account among the most economically important medicinal plants and are commonly used in primary health treatment, where they play a pivotal role in the treatment of various types of diseases via the modulation of biochemical and molecular pathways, besides being a rich source of valuable phytochemicals. In the present review, we summarized the recent advances in botany, phytochemical composition, ethnobotanical uses, food preservation, and the preclinical and clinical efficacy of *Aloe* plants. These data will be helpful to provide future directions for the industrial and medicinal use of *Aloe* plants.

## 1. Introduction

*Aloe* L. is the largest genus in the Xanthorrhoeaceae family, and geographically restricted to Old World [1]. Its name derives from the Arabic word “Alloeh”, meaning “shining bitter substance” [2]. *Aloe* plants have been widely known and used for centuries as topical and oral therapeutic agent due to their health, beauty, medicinal, and skin care properties [2,3]. *Aloe arborescens*, *Aloe barbadensis*, *Aloe ferox*, and *Aloe vera* are among the well-investigated *Aloe* species. Presently, they account among the most economically important medicinal plants and are commonly used in primary health treatment, where they play a pivotal role in the treatment of various types of diseases, through biochemical and molecular pathway modulation [4]. Indeed, *Aloe* plants have been reported for multiple biological properties, including antibacterial and antimicrobial, antitumor, anti-inflammatory, anti-arthritic, anti-rheumatoid, anticancer, and antidiabetic activities, detoxification, treating constipation, flushing out toxins and wastes from the body, promoting digestion (on gastrointestinal disorders treatment), and reducing illnesses likelihood, as well as for immune system deficiencies [3,4,5,6,7,8,9]. Besides, several clinical trials validated the use of *Aloe* gel against several diseases, such as diabetes or antihyperglycemic, wound, and burn-healing topical agents [5,10,11]. The main commercial forms used in the food, cosmetics, and pharmaceutical industries [12,13] consisting of the plant’s fleshy leaves, gel, and latex are pills, jellies, creams, drinks, liquid, sprays, ointments, and lotions [3,13]. The nutrients and phytochemicals that have been identified in *Aloe* plants include vitamins, minerals, enzymes, simple and complex polysaccharides, fatty acids, indoles, alkanes, pyrimidines, aldehydes, dicarboxylic acids, ketones, phenolic compounds, phytosterols, and alkaloids with potential biological and toxicological activities [3,6,9]. On a side note, *A. vera* gel coating has demonstrated postharvest preservative and stabilizing effects in some foods and beverages, and for instance, table grapes [14,15]. However, *A. vera* administration is often related to kidney dysfunction, diarrhea, electrolyte imbalance, and conventional drug interactions. In addition, *Aloe* topical application has been associated with erythema, contact dermatitis episodes, and phototoxicity [3]. *Aloe* fleshy leaves, gel, and latex are the base of the main commercial forms of aloe products (e.g., pills, jellies, creams, drinks, liquids, sprays, ointments, and lotions) [3,13].

In view of the ethnopharmacological uses and pharmacological properties of *Aloe* plants, this review narratively summarized the botany, phytochemical composition, ethnobotanical uses, food preservative effects, and preclinical and clinical efficacy of *Aloe* plants to provide further direction for its utilization in human welfare.

## 2. Habitat and Cultivation of *Aloe* Plants

*Aloe* genus is a monoecious, perennial species with shallow roots. *Aloe* species are mostly inhabitants of arid climates, and are widely distributed in Africa, India, and other arid areas. The largest number of *Aloe* species is approximately 140, and most are found in South Africa [1]. However, they could also be grown in subtropical summer rainfall and winter rainfall regions [16]. The major factors restraining genus distribution are fire tolerance, soil moisture, rainfall, and temperature. *Aloe* species occupy a wide diversity of habitats, varying from sea level to altitudes of 2700 m, and from closed-canopy forests to desert shrub lands. However, some individual species showed particular geography restriction [17], although seed pollinator morphology and specificity also effect *Aloe* species distribution [18].

*Aloe* species can be cultivated in a wide range of soils. The most desirable soil texture is a loamy mixture with pH ranging from 7.0 to 8.5. Nonetheless, some species, such as *Aloe commixta*, *Aloe haemanthifolia*, *Aloe plicatilis*, and most grass aloe species prefer to grow in acidic soils [19]. Temperature requirements for *Aloe* growth range from 4 °C to 21 °C; however, this cold tolerant genus could maintain its growth even below 4 °C. Under optimal environmental conditions, *Aloe* species can reach heights of up to 61–99 cm. Regarding flowering duration, it may vary from May to June [19]. Some *Aloe* species appeared to react to soil mineral composition and produce differently colored flower varieties [20]. Although few *Aloe* genus species require specific pH and mineral composition, the majority of them can thrive in almost any soil type, and require little or no soil preparation before soil planting [21,22]. The ideal soil for establishment after *Aloe* species nursing is well-drained sandy soil or rocky sites. The adult plants of some *Aloe* species (e.g., *A. greatheadii* and *A. secundiflora*) can act as nurse plants themselves, colonizing, and ameliorating harsh conditions in sparsely vegetated or completely denuded landscapes [23,24]. *Aloe* species could also be effective agents for degraded rangeland treatment because of their mate (similar to root systems), which increases soil binding and stabilization [23].

## 3. Phytochemical Composition of *Aloe* Plants

Various species of *Aloe* genus plants have been proven to exert a diverse range of pharmacological activities. In addition, aloes are now considered to be a very interesting source of bioactive compounds [25]. Concurrently, some of the pharmacological activities reported, which support the traditional usages of each aloe species, have also been attributed to the presence of a wide range of phytoconstituents.

*Aloe* plant leaves, which are the most commonly used medicinal parts, are heterogeneous and can be divided into three major parts, namely: (i) the outer green epidermis, primarily consisting of structural components; (ii) the outer pulp region below the epidermis, consisting of vascular bundles where the bitter latex or sap is derived; and (iii) the inner leaf pulp, consisting of aloe gel and containing parenchyma cells. Regarding the different composition of these leaf portions, they are also likely to have distinct classes of bioactive compounds, which is believed to contribute to the different biological properties of leaves [26]. Briefly, the outer green epidermis has been reported to contain anthraquinones, pre-anthraquinones, and their corresponding glycosides [27], while the outer pulp region below the epidermis contains latex that predominantly consists of phenolic compounds, including anthraquinones and pre-anthraquinones, anthrones, chromones, coumarins, flavonoids, and pyrones [28]. On the other hand, the inner leaf pulp contains a high acemannan polysaccharide content, as well as a wide variety of phytochemicals, among them alkaloids, anthraquinones, anthrones, chromones, coumarins, flavonoids, and pyrones [27,29]. Pulp also contains vitamins, minerals, enzymes, and proteins [3]. Indeed, many authors believe that the various biological activities related to different *Aloe* species should be ascribed to a synergistic action between several compounds rather than a single chemical substance [29,30].

### 3.1. General Reports on Aloe Species Phytochemicals

Based on our literature search, various review articles that discuss *Aloe* phytochemical content are mostly focused on *Aloe vera* [2,31], with only Cock [26] providing a review article on the *Aloe* genus, which mainly focused on the close relationship between plants phytoconstituents and antioxidant capacity. However, most of the phytochemicals identified in the genus *Aloe* were not properly associated with the respective *Aloe* species from which they were isolated. Moreover, not all of the phytochemicals were completely cited by Cock [26], although he did cite all of the reports made by original authors. Prior to presenting our own report on *Aloe* species phytoconstituents, we take this opportunity to acknowledge the work of Cock [26], highlighting both bioactive compounds generally isolated and identified from the *Aloe* genus. Indeed, in his article, Cock [26] divided the discussion on *Aloe* genus phytoconstituents into several significant classes, namely anthraquinones, anthrones, chromones, coumarins, pyrans, pyrones, alkaloids, benzene, naphthalene, and furan derivatives.

#### 3.1.1. Anthraquinones of *Aloe* Species

Various types of anthraquinones were also reported to be presented in *Aloe* species leaves, such as aloesaponarin, chrysophanol, and its progenitor prechrysophanol, desoxyerythrolaccin, 1,5-dihydroxy-3-hydroxy methylanthraquinone, helminthosporin, 7-hydroxyaloe emodin, isoxanthorin, laccaic acid-d-methyl ester, nataloe emodin, and its ester nataloe emodin-8-methyl ester, aloechrysone, and aloesaponol. In addition, *Aloe* anthraquinones are often present as *O*-glycosides, such as aloe emodin-11-*O*-rhamnoside, nataloe emodin-2-*O*-glucoside, aloesaponol-6-*O*-glucoside, aloesaponol-8-*O*-glucoside, and aloesaponol-*O*-methyl-4-*O*-glucoside. Other than that, leaf exudates from several *Aloe* species, such as *Aloe saponaria* Haw. and *Aloe elgonica* Bullock were also reported to contain anthraquinone dimers, such as asphodelin and bianthracene, and its glycosylated dimer derivative elgonicardine [26]. The hydroxylated derivatives of aloin, such as 5-hydroxyaloin A, 7-hydroxyaloin, and 10-hydroxyaloin B, as well as their acetate derivatives, 5-hydroxyaloin A 6′-*O*-acetate, 7-hydroxyaloin-6′-*O*-monoacetate, and 10-hydroxyaloin-6-*O*-acetate have also been identified. Except for *A. saponaria* and *A. elgonica*, no other *Aloe* species were reported in the discussion, despite the numerous mentioned anthraquinones isolated from this plant.

#### 3.1.2. Anthrones of *Aloe* Species

Within the anthrones class, barbaloin, which referred to *C*-glycosyl anthrone isomers aloin A and aloin B, was the first *Aloe* anthrone to be isolated and can be detected in nearly 100 *Aloe* species, including *A. vera* and *A. ferox* leaves [26]. In addition, homonataloin and nataloin were consequently isolated from *A. marlothii* Berger. Other anthrones, some of them containing additional rhamnose, cinnamic, or coumaroyl moieties, have also been isolated from various *Aloe* species leaves, which include aloinoside, aloe barbendol, aloe-emodin anthrone, chrysophanolanthrone, aloe emodin-10-C-rhamnoside, 8-*O*-methyl-7-hydroxyaloin, 6′-*O*-cinnamoyl-8-*O*-methyl-7-hydroxyaloin, 6′-*O*-*p*-coumaroyl-7-hydroxyaloin, 7-hydroxyaloin-4′,6′-*O*-diacetate, 6′-*O*-cinnamoyl-5-hydroxyaloin A, microstigmin A, deacetyllittoraloin, littoraloin, littoraloside, microdontin, and homonataloside [26].

#### 3.1.3. Chromones of *Aloe* Species

Another phytochemical group, known as chromones, are the most abundant phenolic compound class in *Aloe* leaves [26]. Aloeresin A and aloesin (previously known as aloeresin B), as well as 2′-*p*-*O*-methlcoumaroylaloesin, have been classified as some of the most commonly found *Aloe* leaf constituents. In addition, several isomeric and substituted isomeric forms, including aloeresin C, aloeresin D, aloeresin E, aloeresin F, iso-aloeresin A, and iso-aloeresin D have also been reported. These were later followed by the successful identification of a wide variety of chromones from distinct *Aloe* species, including methylated derivatives, such as 7-*O*-methylaloesin, 7-*O*-methylaloesinol, 7-*O*-methylaloeresin A, 8-[*C*-*B*-*D*-[2-*O*-(*E*)-cinnamoyl]glucopyranosyl]-2-[(*R*)-2-hydroxypropyl]-7-methoxy-5-methylchromone, 8-*C*-glycosyl-7-*O*-methylaloediol, 8-*C*-glycosyl-7-*O*-methyl-*S*-aloesol, 2-acetonyl-7-hydroxy-8-(2-furanonyl)-7-hydroxy-5-methylchromone, and 7-hydroxy-2,5-dimethylchromone. In addition to these, chromones containing cinnamic and coumaroyl moieties, such as 8-*C*-glycosyl-(2′-*O*-cinnamoyl)-7-*O*-methyl-aloediol, 8,2-acetonyl-8-(2′,6′-di-*O*,*O*-coumaroyl)-glucopyranosyl-7-hydroxy-5-methylchromone, 2-acetonyl-8-(2′,cinnamoyl)-glucopyranosyl-7-hydroxy-5-methylchromone, 6′-*O*-coumaroylaloesin, and 2′-*p*-*O*-methlcoumaroylaloesin have also been identified [26]. Unfortunately, for any of the chromones cited, not one specific *Aloe* species was excerpted.

#### 3.1.4. Coumarins, Pyrans, and Pyrones of *Aloe* Species

Feralolide and dihydroisocoumarin glycoside are coumarins that have been identified in *A. ferox* and *A. hildebrandtii* [26], respectively. Pyrans (e.g., bisbenzopyran) and pyrones (e.g., aloenin, aloenin aglycone, aloenin acetal, aloenin B, and aloe-2″-*p*-*O*-coumaroyl ester) have also been identified in several *Aloe* species leaf exudates [26]. Except for *A. ferox* and *A. hildebrandtii*, the other *Aloe* species, from which coumarins, pyrans, and pyrones were isolated, were not revealed.

#### 3.1.5. Alkaloids of *Aloe* Species

Several alkaloids have been isolated from several *Aloe* species [26]. *N*-methyltyramine and *O*,*N*-dimethyltyramine have been reported as being the most common *Aloe* alkaloids, whereas γ-coniceine was only found in a few species. In contrast, coniine has only been reported to occur in one species, *Aloe viguieri* Perrier [26]. Except for *A. viguieri*, the other *Aloe* species, from which the alkaloids were identified, were not quoted.

#### 3.1.6. Benzene, Naphthalene, and Furan Derivatives of *Aloe* Species

Several benzene, naphthalene, and furan-based bioactive compounds have also been identified as common constituents of *Aloe* plants [26]. Among the identified benzene derivatives are protocatechuic acid, methyl-*p*-coumarate, and pluridone, which have been identified in several *Aloes*. Fluridone, which is the only sulfur derivative containing the benzene derivative identified from *Aloe* plants, was identified from *A. pluridens* Haw. Various naphthalene derivatives, namely aglycone isoeleutherol, isoeleutherol-5-*O*-glucoside, feroxidin, feroxidin A, feroxidin B, and plicataloside, have been isolated from *Aloe* plants, with aglycone isoeleutherol and isoeleutherol-5-*O*-glucoside specifically detected from *Aloes* roots portions. In addition to these, several of the naphthalene compounds, such as 5-OH-3-methylnaphto[2,3-c]furan-4(1*H*)-one, 3-methylnaphto[2,3-c]furan-4(9*H*)-one, and 3-methylnaphto[2,3-c]furan-4,9-dione have been found to contain a furan moiety [26]. Except for *A. pluridens*, the other *Aloe* species, from which the benzene, naphthalene, and furan derivatives were identified, were not named.

#### 3.1.7. Flavonoids of *Aloe* Species

Several flavonoids were also detected in *Aloe* plants, with only naringenin, apigenin, isovitexin, and dihydro-isorhamnetin cited as being the major ones [26]. Nevertheless, the actual *Aloe* plants, from which flavonoids have been isolated, were not cited.

#### 3.1.8. Sterols of *Aloe* Species

Phytosterols, such as cholesterol, campesterol, β-sitosterol, and lupeol together with their glucosides have also been cited to be present in *Aloe* leaves [26]. However, the actual *Aloe* plants, from which the sterols were isolated, were not mentioned.

#### 3.1.9. Other Phenolic Constituent of *Aloe* Species

Although a number of other secondary metabolites were claimed to be present in *Aloe* leaves, Cock [26] only specifically cited the presence of tannins. Moreover, the actual *Aloe* plants, from which the secondary metabolites were isolated, were not indicated.

#### 3.1.10. Non-Phenolic Components of *Aloe* Species

Polysaccharides are the non-phenolic components that are present in high abundance in *Aloe* leaf gels [26]. However, their presence within *Aloe* leaves are variable, and there is huge diversity among the different individual *Aloes*. According to Femenia et al. [32], polysaccharides composition and concentration also change with seasonal variations and growing environment conditions. Although several polysaccharides were detected in *Aloe* species, Cock [26] only mentioned acemannan (a long chain polymer of β (1→4) linked galactomannan saccharides). Unfortunately, the exact species from which polysaccharides were isolated were not mentioned.

#### 3.1.11. Vitamins of *Aloe* Species

Cock [26] also cited the presence of various vitamins, namely vitamin C (ascorbic acid), vitamin B1 (thiamine), vitamin B2 (riboflavin), vitamin B6 (pyridoxal phosphate), vitamin B12 (cyanocobalamin), and vitamin E (α-tocopherol) in unspecified *Aloe* leaf gels.

#### 3.1.12. Mineral Nutrients in *Aloe* species

Moreover, Cock [26] also mentioned that leaf gels from several *Aloe* species contain inorganic minerals, including magnesium, zinc, calcium, potassium, sodium, iron, phosphorous, manganese, copper, and molybdenum. However, species were not mentioned.

### 3.2. Specific Reports on Aloe Phytoconstituents

Cock [26] claimed that his review article on *Aloe* genus is not a comprehensive report. This claim was further supported by the lack of information with regard to *Aloe*’*s* phytoconstituents, such as no information on: (i) types of *Aloe* parts or extracts used to isolate bioactive compound(s); (ii) *Aloe* plants species used in the isolation of bioactive compound(s); and (iii) the location from which *Aloe* plants were collected, to name a few. Moreover, several phytoconstituents that were reported in some articles were not mentioned at all by Cock [26]. Taking these issues into consideration, the present review was performed with an attempt to provide more detail with regard to *Aloe* genus-isolated phytoconstituents.

#### Phytochemical Studies on *Aloe* Species

Many reports have been published concerning *Aloe* phytochemicals. Due to their economic and medical importance, *Aloe* phytoconstituents, especially those from the *A. arborescens*, *A. barbadensis*, *A. ferox*, and *A. vera* species, have been intensively investigated. The summary of phytochemical studies on *Aloe* species, including plant parts, principal constituents, and corresponding literatures is represented in Table 1, Table 2, Table 3, Table 4 and Table 5, according to investigated extraction methods. In most cases, leaf *Aloe* plants exudates were collected and used for phytoconstituent investigation purposes. Some researchers used fresh plant materials. *Aloe* plant extract chemical compositions were analyzed by solvent fractionation, column chromatography, preparative thin-layer chromatography (TLC), high-performance liquid chromatography (HPLC), HPLC–mass spectrometry (MS), and gas chromatography–mass spectrometry (GC–MS). In general, around 20 chemical constituents, including aloin A, aloin B, aloinoside A, and aloinoside B, aloesin, plicataloside, isovitexin, and aloe emodin were reported as principal *Aloe* species components.

**Figure 1:**
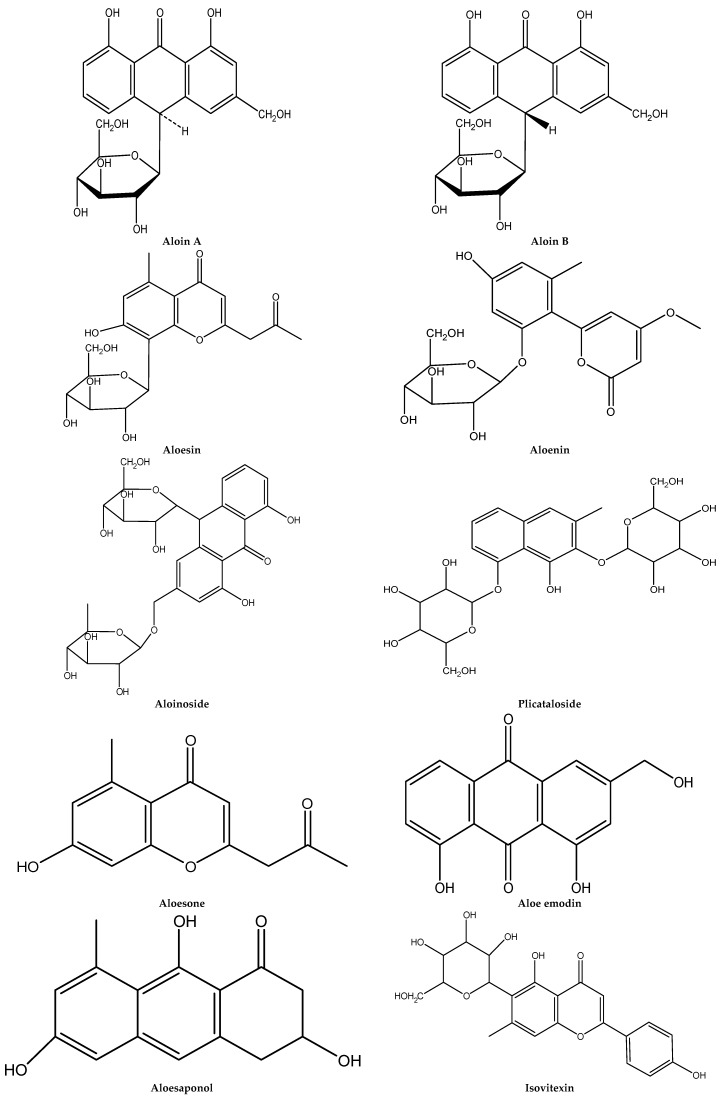
The chemical structures of the main phytochemicals of *Aloe* species.

The chemical structure of main *Aloe* species phytochemicals is represented in Figure 1.

## 4. Traditional Medicine Use of *Aloe* Plants

This section summarizes the traditional use of *Aloe* plants for treating various ailments from different parts of the world. Among 446 *Aloe* species, belonging to the Xanthorrhoeaceae family, *Aloe vera* (L.) Burm. f., also known as *A. barbadensis* Mill., is the most commonly used species in traditional medicine in Indian subcontinent. Other species reported to be used are *A. arborescens*, *A. littoralis*, and *A. pirottae*. Akaberi et al. [95] provided the most recent review of the *Aloe* species’ therapeutic effects in traditional and modern medicine. Gastrointestinal activities, hepatoprotective properties, and beneficial effects against skin problems, such as wounds, injuries, and infective diseases, are among the most frequently reported *Aloe* species properties [95].

Most of the *Aloe* plants usage reports are documented from Asia (India and Nepal), and a few exist from other parts of the world, mostly from Africa through ethnomedicine studies. *A. vera* is commonly called Ghiu kumari in India and Nepal.

As previously introduced, the most widely used part of *Aloe* is the leaf. The most commonly used part is leaf gel, which is effective for treating cuts and burns, gastrointestinal disorders, and maintaining blood pressure. Jelly obtained from the leaf is used to treat cuts, burns, and other skin complaints in the Indian subcontinent [96,97,98,99,100]. It is also eaten by people suffering from high blood pressure, gastritis, and stomach-related diseases [96]. In Sikkim-India, *A. vera* is used for its antihyperglycemic effect [101]. The *A. vera* purgative property is also documented in the Sikkim government database [99].

Sushen et al. [102] have documented the extensive uses of *A. vera* in the traditional health care system in India. They report that juice preparation is used for sunstroke; leaf gel is useful to treat gastric intestinal problems, such as indigestion, candidiasis, constipation, diarrhea, colitis, and digestive issue relief, such as heartburn and irritable bowel syndrome. Chewing *A. vera* leaf or massaging on gums with an index finger gently using its gel will cure bleeding gums and toothache; it can also be used to treat menstrual cramps; gel mixed with a pinch of patika (alum, potassium aluminum sulfate) and put on a cloth can be put on eyes to cure eye problems. Leaf gel has several use reports. Leaf gel powder is mixed with raw turmeric juice, and a few drops is put into the ear and nose three to four times a day to reduce infections. Leaf gels are also mixed with a pinch of dried turmeric powder, a pinch of powdered dried seeds of bitter gourd, and a little sugar to make it sweet, and taken two times a day. It is also mixed with a few drops of raw garlic juice and a pinch of dried turmeric powder to make a homogeneous paste, and two to three spoons are taken three times a day for diarrhea; gel is used for uterine cancer and cervical cancer; it is also used for constipation, rectal infection, and ulcers. Several other leaf gel uses have been documented, and some other *A. vera* uses include: lowering cholesterol in blood; for miscarriage and anti-abortive treatment; treating arthritis, joint pains, body pains, and muscle pain; increasing potentiality and sperm count; treating heart disease, depression, stress release, anger management, stability management, and so on. Also reported is for cuts and wounds, white hair reduction, hair fall, ticks, mites, dandruff, baldness, grey hair, dry split hair, etc. In India, it is also used for piles, lower abdomen pain, migraine, breast pain, tuberculosis, stomach ulcers, heartburn, indigestion, dysentery, sexually transmitted infections, endometriosis, fibroids presence in uterus, gonorrhea, etc. Pimples and achiness are also treated with *A. vera*. Protection from ultraviolet (UV) irradiation, post-operative care, stretch marks, learning and memory improvement, cancer, diabetes, hepatitis, AIDS, and weight loss are other uses of *Aloe* leaf gel.

Leaves are also chewed to cure skin and uterine disorders and treat jaundice in Nepal [103,104,105]. In India, leaves are used for stomachic, tonic, purgative and anthelmintic purposes; juice is put on the head in high fever to reduce body temperature [106]. Leaf juice is also applied on fresh burns. Juice is also given to diabetic patients and for urinal troubles [105]. The Limboo tribe of Sikkim use leaf juice on burnt wounds, which also helps cool pain [107]. Whole plant juice, pulp, or paste is used as a remedy in intestinal worms in children, as well as a hepatic stimulant, stomachic, and liver and spleen enlargement [108].

Leaf juice is given for a stomachache and as a tonic [109]. Juice is also commonly used in case of constipation [109,110]. Leaf juice given in case of indigestion, hemorrhoids, and peptic ulcers [111]. Juice is also used in rheumatic pains, fever, jaundice, menstrual disorder, suppression, and gonorrhea, and juice is applied locally for burns and skin irritation [101].

Leaf pulp is also taken to treat dysentery [112] and other stomach disorders in Nepal [113]. It is used to cure burn wounds and regulate menstruation, constipation, and ascariasis [99]. The use of *A. vera* as an emmenagogue is also reported from Panchthar Nepal [97]. Leaf is used in catarrh, cough, and overheating problems [114], and *Aloe* species leaf gel, including *A. vera*, is also used for diabetes [115,116,117].

*A. vera* is also used to treat fresh and bleeding or infected wounds, burns, eczema, and dandruff in Mexico [118]. It is also used to treat HIV in South Africa [119,120]. Pulp and juice obtained from *A. arborescens* is used on dermatosis and against articular pains in Italy [121].

*A. littoralis* is used to treat *Bilharzia* in the traditional health care system of Namibia [122]. Another species reported to be used in traditional medicine from Africa is *A. pirottae*, but its detailed use is not mentioned [123].

## 5. Food Preservative Applications of *Aloe* Plants

Nowadays, the food industry looks for new sources of natural compounds with different properties [124,125,126,127,128,129,130,131,132]. It is considered that, through the plant kingdom, *Aloe* species, especially *A. vera*, is one of the most applied medical plants worldwide [133]. These plants have been used in folk medicine from different therapeutic purposes, due to its purgative effect, for skin disorder healing and beauty treatments. Numerous reports show that the *Aloe* leaf possesses a wild spectrum of activities, including, anticancer, antioxidant, anti-inflammatory, immunomodulatory, hepatoprotective, antiulcer, and antidiabetic as well as found application in dermatology, to treat radiation-caused skin conditions and in gastroenterology or gynecology as a bactericidal, viricidal, or fungicidal. The widespread use of this plant is the result, *inter alia*, of the content of approximately 200 biologically active compounds, as previously introduced, characterized by a synergistic effect. In cosmetology, *Aloe* species are used in creams, soaps, and shampoos production. Not least, industrial applications of these plants include beverages, ice cream, food supplements, and others [16,30]. Despite the industrial use of plants belonging to the *Aloe* genus, previous studies of antimicrobial properties are mainly directed toward isolates obtained from human skin. Undoubtedly, these plants are characterized by their natural antimicrobial potential, and their use in food production.

## 6. Antimicrobial Activity

### 6.1. Antibacterial Activity

In vitro studies have shown that *A. vera* is characterized by activity against Gram-negative and Gram-positive bacteria. Petroleum ether, dichloromethane, and water extracts of upper stem, young bark, mature bark, leaves and roots of *A. barberae* from South Africa were evaluated for their antimicrobial activity against Gram-positive (*Bacillus subtilis*, *Staphylococcus aureus*) and Gram-negative (*Escherichia coli*, *Klebsiella pneumoniae*) bacteria [71]. Petroleum ether and dichloromethane extracts of mature bark, leaves, and roots exhibited significant activity against all bacteria, with minimum inhibitory concentrations (MIC) ranging from 0.195 mg/mL to 1.56 mg/mL. In another study, the authors evaluated the antimicrobial properties and phenolic contents of medicinal plants used by the Venda people. It was found that *A. chabaudii* roots exhibited low levels of phenolic compounds as also weak antimicrobial activities against *B. subtilis*, *S. aureus E. coli*, and *K. pneumoniae* [134]. Generally, it is believed that *A. vera* acetone extracts exhibit stronger activity against *S. aureus*, *Streptococcus pyogenes*, *Pseudomonas aeruginosa*, and *E. coli* compared to aqueous or ethanol extracts. Lawrence et al. [67] documented the antibacterial property of *A. vera* gel extracted using different solvents. They found differences between the extract activities against *S. aureus*, *S. pyogenes*, *B. subtilis*, *E. coli*, *Ps. aeruginosa*, *K. pneumoniae*, *Salmonella typhi*, and *Bacillus cereus*. In general, the results of the agar well diffusion method showed that the inhibition zones ranged from 12.66 mm (*E. coli*) to 23.33 mm (*B. cereus*). On the other hand, methanol extract exhibited the strongest activity against *B. cereus* (22.33 mm) followed by *S. pyogenes* (15 mm), and the least for *S. typhi* (9.66 mm). Finally, according the results obtained in their research, acetone extract gave the lowest values of inhibition zones, ranging from 6.00 mm (*E. coli*) to 7.33 mm (*S. pyogenes*). What is more, no differences between acetone and controls were noted for *P. aeruginosa* and *S. typhi*. In general, it is well noted that plant extracts show greater antibacterial activity against Gram-positive than Gram-negative bacteria [135]. Also, in the study of Ferro et al. [136], Gram-positive bacterium *S. pyogenes* was more susceptible to *A. vera* gel than Gram-negative *Shigella flexneri*. The effective growth inhibition was achieved with aloe concentrations of more than 100 mg/mL for *S. flexneri* and 25 mg/mL for *Streptococcus pyogenes*. The authors found that sap extract was more effective than leaf extract against *E. coli*, *B. subtilis*, *S. aureus*, and *P. aeruginosa*. Sap water extract (100 µg/mL) showed the strongest inhibitory properties against *B. subtilis* and *P. aeruginosa*. Jonson et al. [137] studied antibacterial activity of leaf extract from *Aloe vera*, *Datura stromonium*, *Pongamia pinnata*, *Lantona camara*, and *Calotropis procera*. They found that from all of the tested alcoholic and aqueous extracts, *A. vera* showed the strongest activity against *E. coli* and *S. aureus*. What is more, aloe polysaccharides were used in tea tree or palmarosa essential oil combinations as a natural strategy against *Xanthomonas fragariae* (bacterial angular leaf spot disease, which is an important strawberry disease responsible for significant yield losses) [138]. It was found that these preparations reduce disease severity and activate plant defenses, and that *Aloe* polysaccharides alone reduced *X. fragariae* growth by up to 44%. The authors concluded that tested essential oils and polysaccharides from aloe can be considered as potential agents for plant disease control and could play a significant role in product formulation for strawberry leaf spot control. The essential oils into aloe polysaccharide preparation contributed more effectively to reduce the disease severity, either by its antimicrobial activity or by the plants’ defense mechanism activation. The antimicrobial activities of *A. vera* juice were tested in the work of Alemda and Agaoglu [139].

In the work of Dharajiya et al. [140], *A. barbadensis* leaf extract antibacterial activity was evaluated against *E. coli*, *P. aeruginosa*, *B. cereus*, and *Serratia marcescens*. They found maximum inhibitory activities against *S. marcescens* (hexane extract) and *B. cereus* (methanol extract). In general, the authors noted that methanol extract showed an inhibitory effect against all of the tested bacterial strains, while ethyl acetate extract showed no inhibitory activities. Similar results of antibacterial activity against *E. coli*, *B. subtilis*, *S. epidermidis*, and *S sonnei* were obtained by Coopoosamy and Magwa [79]. They found that the MIC of emodin and aloin A ranged from 62.5 mg/mL against *B. subtilis* and *E. coli* to 250 mg/mL against *S. epidermidis* and *S. sonnei* [79,141]. *A. vera* sap and leaf extracts were investigated for antimicrobial properties by Abakar et al. [142]. The disc diffusion method revealed that juice inhibits *Mycobacterium smegmatis*, *K. pneumoniae*, *Enterococcus faecalis*, *Micrococcus luteus*, and *Bacillus sphericus* growth. They concluded that the juice obtained from aloe can be used for antimicrobial activity in cosmetics, pharmacy, and the food industry. *S. mutans* growth inhibition was subject of investigation by Jain et al. [143]. The authors used crude, organic solvent-based and aqueous extracts from *A. vera* leaves, neem (*Azadirachta indica*), tulsi (*Ocimum tenuiflorum*), amla fruits (*Emblica officinalis*), garlic cloves (*Allium sativum*), and ginger rhizomes (*Zingiber officinale*). The MIC results determined by the agar well diffusion method showed that 25 mg/mL of organic solvents extract inhibited tested bacterium growth. On the other hand, the MIC values for aqueous extract equaled 50 mg/mL. It is worth noting that the organic solvent extract activity from aloe was comparable to garlic and alma. The antibacterial activity of polysaccharides from *Aloe* spp. has been attributed to phagocytic leucocytes stimulation to destroy bacteria. *A. vera* compounds with particular antimicrobial activity are saponins, acemannan, and anthraquinones derivatives [144]. *Aloe*-emodin effect on *Helicobacter pylori* N-acetyltransferase activity showed dose-dependent inhibition [145]. Cellini et al. [146] attributed *H. pylori* inhibition to the polysaccharides that are present in gel, exhibiting an anti-adhesive effect. *A. vera* aqueous extract effect on *E. coli* morphological and physiological properties were described by Kargaran et al. [147]. They found that the aloe extract MIC value equaled 2.23 mg/mL. What is more, another aloe plant—*A. ferox*—is known to show activity against wild bacteria. *A. ferox*-isolated compounds (aloe emodin, chrysophanol, and aloin) activity were investigated by Kambizi et al. [56]. They found that aloe emodin and alonin A exhibit inhibitory activities against *B. cereus*, *B. subtilis*, *S. aureus*, *E. coli*, *Staphylococcus epidermidis*, and *Shigella sonnei*. Chrysophanol was characterized by weaker antibacterial action, inhibiting *B. subtilis*, *S. epidermidis*, and *E. coli* strains. On the other hand, pyrocatechol, 2-vinyl crotonaldehyde, ascorbic acid, *p*-coumaric acid, and cinnamic acid isolated from plant have shown a wide spectrum of antibacterial activity [148].

Radi et al. [149] evaluated the effect of gelatin coating incorporated with *A. vera* gel and green and black tea extracts on the physicochemical, microbial, and sensorial properties of fresh-cut oranges stored at 4 °C for 17 days. They noted that coating materials with gelatin incorporated with *A. vera* and green tea extracts successfully retarded microbial growth and extended shelf life during storage. Chen et al. [150] showed that the antimicrobial activities of composite films increased as the amount of aloe gel powder used in composite films increased. The average area of inhibitory zones of *Citrobacter freundii*, *Escherichia coli*, *Enterobacter aerogenes*, *Serratia marcescens*, *S. aureus*, and *B. cereus* for films with aloe⁄gelatin (1:4) and aloe⁄gelatin (4:1) compositions were 1.63 ÷ 2.38 mm and 3.82 ÷ 4.80 mm, respectively. *A. vera* potential application as an edible coating was provided by Benítez et al. [151]. They found significant microbial population reduction in fresh-cut kiwifruit treated with 15% *A. vera* during 11 days of storage. The results at the last day of analysis were 4.97 log CFU (colony-forming units)/g for 15% aloe and 5.75 log CFU/g for control. What is more, they found that *A. vera* can be used as a coating to both extend postharvest shelf life and maintain product sensory properties through the storage period [152]. Similar results were obtained by Sogvar et al. [153] on aloe coatings that have been used to maintain the quality of postharvest strawberry fruits. The application of aloe and 5% of ascorbic acid reduced the mesophilic bacteria population from 3.63 log CFU/g for control to 3.13 log CFU/g.

### 6.2. Antifungal Activity

*Aloe* species’ availability, safety, and bioactivity make them an interesting alternative as control agent used in preharvest and postharvest fungal diseases of fruits and vegetables. Studies showed that *A. vera* reduces *Penicillium*, *Botrytis*, and *Alternaria* spore survival by up to 20% [154], as well as inhibits *Fusarium*, *Rhizoctonia*, and *Colleotrichum* mycelium growth by up to 38% [155]. What is more, in the work of Castillo et al. [156], it was reported that *A. vera* gel inhibits *Penicillium digitatum* and *Botrytis cinerea*. *A. vera* gel was analyzed as an antifungal agent against six fungi causing plant diseases: *Fusarium oxysporum*, *Alternaria alternate*, *Colletotrichum gloeosporioides*, *Bipolaris spicifera*, *Curvularia hawaiiensis*, and *Botryotinia fuckeliana*. It was found that gel was most effective against *F. oxysporum* [157]. The authors found that films with the highest *A. vera* ratio were effective in controlling fungal contamination. Nectarine treated with *A. vera* gel alone, or with the addition of thymol, inhibited the fungal growth of inoculated *Rhizopus stolonifer*, *Botrytis cinerea*, and *Penicillium digitatum*. Therefore, *Aloe* application led to a significantly lower fungal infection (two to threefold) than in non-treated nectarines. It was found that the addition of thymol did not generally improve the aloe gel efficacy to reduce infection [158]. In the work of Vieira et al. [159], chitosan and *A. vera* liquid fraction coatings presented the best uniformly coat blueberry surface characteristics. The authors showed that the microbiological growth of *Botrytis cinerea* was reduced by 42% in coated blueberries after 25 days. Overall, coatings extended the shelf life of blueberries for five days (compared with the control sample), demonstrating that a chitosan and *A. vera* combination shows high potential in expanding shelf life. The antifungal activity from eight *Aloe* species gels (*A. arborescens* Mill., *A. aristata* Haw., *A. claviflora* Strydenburg, *A. ferox* Mill., *A. mitriformis* Mill., *A. saponaria* Ait., *A. striata* Haw., and *A. vera* L.) were evaluated against *B. cinerea*, *P. digitatum*, *Penicillium expansum*, and *P. italicum* [160]. The authors concluded that antifungal activity was higher for *A. ferox*, *A. mitriformis*, and *A. saponaria* than *A. vera*, which can be correlated with aloin content. Nidiry et al. [161] have also reported that aloin and aloe-emodin from *A. vera* could be important antifungal moieties. Guillén et al. [160] reported that *A. arborescens* could be even more effective than *A. vera* gel for preservative purposes in edible coatings, affecting climacteric fruit quality. *A. vera* and green tea extracts used in gelatin-based edible coating for fresh-cut oranges reduced the total fungal count [149]. *A. vera* coatings effectively controlled or inhibited fungal populations during strawberry storage, and therefore was considered an effective natural agent against bacteria and yeasts-associated postharvest diseases [153]. The total number of yeasts and molds obtained for fresh-cut kiwi samples coated with aloe were about 10 times lower than for the control sample. Additionally, an *A. vera* coating maintained fruit firmness, and prevented ascorbic acid losses and yellowing due to ripening. In contrast, fruit treated with an alginate-based coating had higher microorganism counts than the control samples [152]. Benítez et al. [151] studied the efficacy of an edible coating based on *A. vera* gel at different concentrations: 1% (*v*/*v*), 5% (*v*/*v*), and 15% (*v*/*v*). They found that an aloe coating reduced respiration rates and microbial spoilage in sliced kiwi fruit. After seven days of storage, yeast and molds load dropped by approximately one logarithmic unit for slices coated with 15% and 5%. Similar findings were stated by Martínez-Romero et al. [162]. In their work, an *A. vera* gel coating maintained ready-to-eat pomegranate arils. The authors used different aloe concentrations, as well as aloe plus ascorbic acid, and found that *A. vera* treatments led to significantly lower counts of both mesophilic aerobics, and yeasts and molds. What is more, aloe coatings led to firmness retention and increased the total anthocyanins and total phenolics levels. The antifungal effects of 2% (*w*/*v*) *A. vera* were also evaluated against *Colletotrichum gloeosporioides* on avocado fruit by Bill et al. [163]. According to the results, aloe showed weaker properties than thyme oil with chitosan combination. *A. vera* gel was evaluated for their antifungal activity in the study of Sitara et al. [164]. The authors noted that 0.35% of tested gel completely inhibited plant pathogenic fungal growth for *Alternaria alternata* and *Drechslera hawaiensis*. In comparison with the control medium, significant growth inhibition was also found in *Aspergillus niger*, *A. flavus*, and *P. digitatum*. Hassanpour [165] found that coating materials containing *A. vera* gel reduce fungal decay of raspberry fruits (*Rubus* spp.) during eight days’ incubation at 4 °C, without differences between different gel levels. Functional films with *A. vera* gel were noted to increase different papaya fruits (*Carica papaya*) shelf life during 15 days of storage [166]. The authors found that *A. vera* gel significantly inhibited papaya fruits ripping. Generally, *A. vera* use as a functional film component was also noted for grapes (*Vitis vinifera*) [167], pineapple (*Ananas comosus*) [168], or tomatoes [169] as well as cherry tomatoes [158].

In other studies, *A. ferox* methanol extract showed activity against *Candida albicans* with an MIC value of 20 mg/mL, while the MIC of aloin used against these yeasts equaled 5 mg/mL. On the other hand, *Aloe ferox* acetone extract was shown to exhibit fungicidal activity at 10 mg/mL against five fungal strains: *Alternaria alternata*, *A. niger*, *Mucor hiemalis*, *Penicillium notatum*, and *Schizophyllum commune* [170]. Abakar et al. [142] noted that sap water extract and *A. vera* leaves exhibited intermediate susceptibility against *A. niger* and *C. albicans*. Subramanian et al. [171] found that *A. vera* leaf gel ethanol extract showed strong activity against *Aspergillus fumigatus*, *A. niger*, *A. flavus*, *Fusarium oxysporum*, and *Microsporum canis*. On the other hand, *A. vera* fresh leaf hydroalcoholic extract showed antifungal activity against *Botrytis gladiolorum*, *Fusarium oxysporum*, and *Penicillium gladioli* mycelial growth [172]. *A. vera* gel and leaf extract activity against *Trichophyton mentagrophytes*, *T. schoeleinii*, *M. canis*, and *Candida albicans* were evaluated by Olaleye et al. [173]. In this research, only gel inhibited *T. mentagrophytes* growth, while leaf extract possessed inhibitory effects on *C. albicans*. The antifungal activity against *Aspergillus niger*, *A. flavus*, *Aspergillus oryzae*, *Penicillium chrysogenum*, and *Trichoderma viride* were evaluated by Dharajiya et al. [140]. Maximum inhibitory activity was found for aqueous extract against *A. niger*. On the other hand, methanol extract showed weak inhibitory activity against *A. oryzae*. Two extracts (hexane and ethyl acetate) failed to express antifungal activity against any of the fungal strains used in the study. What is more, the authors found that *P. chrysogenum* and *T. viride* were found to be resistant to the tested extracts. Das et al. [148] have reported that a protein isolated from *A. vera* shows antifungal activity against *Candida* species, specifically *C. paraprilosis*, *C. krusei*, and *C. albicans*. Sequencing analysis showed the isolate to be a lectin-like protein that inhibits trypsin, revealing a protease inhibitory function.

## 7. In Vitro and In Vivo Biological Activities of *Aloe* Plants

In the following subsections, different in vitro and in vivo *Aloe* plant species biological effects are carefully described and briefly resumed in Table 6.

### 7.1. Wound Healing and Cell Proliferation

*A. vera* has been used for the treatment of skin damage in several cultures [174]. In vitro extracts of *A. vera* stimulated several cell type proliferations. In many researches, treatment with whole *A. vera* gel extracts resulted in faster wounds healing [175,176]. The mannose 6-phosphate present in *A. vera* gel is considered to be the active ingredient for wound healing [177]. Mannose 6-phosphate heals wounds by increasing cell phagocytic activity [178]. It is thought that mannose 6-phosphate increases the wound area contraction rate [179] and collagen synthesis [180]. During wound healing, the polysaccharides that are present in *A. vera* induce fibroblasts proliferation and hyaluronic acid and hydroxyproline production, which play an important role in extracellular matrix remodeling [5]. A class of plant growth regulators, gibberellins, which are available in *A. vera*, also enhance collagen and elastin formation for breaking strength improvement, by interfering with a collagen cross-link for wound contraction, reducing wrinkle formation [2,181,182,183]. Polysaccharides and glycoproteins isolated from plants were reported to have wound-healing activity [184,185]. The wound-healing activity of saponin that is present in *A. vera* was also reported [186]. Acemannan is considered to be a main functional component of *A. vera*; it is composed of a long chain of acetylated mannose [33,135,184]. Indeed, acemannan stimulates wound healing and hard tissue regeneration by inducing cell proliferation [187]. Eight *Aloe* species (*A. arborescens*, *A. brevifolia*, *A. eru*, *A. ferox*, *A. grandidentata*, *A. perfoliata*, *A. saponaria*, and *A. vera*) also provided significant accelerating effects on diabetic wound healing in rats following the topical application of leaf methanol extracts [36].

### 7.2. Intestinal Absorption and Purgative Action

*Aloe* products have been used for drug absorption enhancement with low bioavailability due to extensive efflux [188]. Carien et al. [188] demonstrated an increase in drug permeability in the presence of *Aloe vera* gel and whole leaf materials. This is thought to be attributed to the opening of tight junctions by *A. vera* gel and precipitated polysaccharides. Laxatives are substances that loosen stools. *Aloe* is also used as a laxative due to its ability to reduce intestinal water absorption. Anthraquinones that are present in plants act as potent laxatives through mucous secretion stimulation, thereby increasing intestinal water content [189]. Active anthraquinones, such as aloin, aloe-emodin, and emodin are linked to aloe purgative action [5,190]. Aloin, which is present in gel, can be metabolized by colonic flora to reactive aloe-emodin, which is responsible for purgative activity [5,133]. Five phytosterols that are present in *A. vera* gel are able to reduce visceral fat accumulation, and influence glucose and lipid metabolism in animal model experiments. They also reduced large-sized intestinal polyps [5]. Aloe-emodin, emodin, and rhein synergistically exerted a potentiating purgative effect on mice [191]. *A. vera* laxative effect was shown after 6–24 h or more from oral anthraquinone administration [192].

*A. ferox* is widely used for its potent laxative and cathartic effects, which are attributed to anthraquinones and in particular to aloe-emodin [193]. *A. ferox* leaf water extract displayed in vivo laxative effects via improved intestinal motility in loperamide-induced constipated rats [194].

In addition to its laxative properties, *Aloe* could strengthen the stomach, and was traditionally used as a carminative and appetizer agent. It has also been suggested for relieving stomach pain [95]. *Aloe* was known as a good remedy for hemorrhoids and anal disorder treatment. Clinical and experimental studies have shown that *Aloe* preparation administration is useful for a wide range of gastrointestinal problems. *Aloe* extract and a number of its compounds have been shown to ameliorate inflammation and improve clinical and histopathological colitis symptoms in animal models. Significant antiulcer and gastroprotective activities were also observed after administration of *Aloe-*containing preparation [95]. *Aloe* is thought to be a potential agent in treatment of gastrointestinal cancers [195,196].

### 7.3. Anti-Inflammatory and Immunomodulatory Effects

Cyclooxygenase (COX) enzymes (also known as prostaglandin-H_2_-synthases) act as catalysts in the production of prostaglandins (highly active pro-inflammatory mediators) from arachidonic acid (hydrolytically released from membrane phospholipids) during inflammatory processes [197]. Prostaglandin production inhibition by inhibiting COX enzymes, particularly the COX-2 enzyme (an isoform induced under pathological conditions) is one of the mechanisms of action of some non-steroidal anti-inflammatory drugs (NSAIDs) used for the symptomatic treatment of inflammation. Although different levels of activity (against COX-1 only) were reported in 51 different *Aloe* species [198], COX-1 isoform inhibition is not desirable, because this enzyme is largely known to be constitutively expressed in most tissues for maintaining some physiological functions [199]. *Aloe* administration has been demonstrated to result in phagocytic and proliferative activity raise by inhibiting COX pathways and reducing prostaglandin E2 production [200,201]. Lindsey et al. [202] reported that *A. ferox* methanol extract exhibited COX-1 inhibitory effects. Albumin transcription levels and tumor necrosis factor (TNF)-α genes are involved in the early phase of acute inflammatory response. In rats treated with aloe-emodin, an abolishment of albumin gene transcription was observed. TNF-α was weakly detectable in livers after aloe-emodin administration. Histological analysis showed a reduced inflammatory infiltration of the lymphocytes and Kupffer cells observed in rats treated with aloe-emodin [203]. *A. vera* can inhibit the inflammatory process following burn injury by reducing leukocyte adhesion and pro-inflammatory cytokine production [204]. *Aloe* polysaccharide pretreatment can attenuate cerebral ischemia and reperfusion injury in severe traumatic–hemorrhagic rats, through inhibiting systemic inflammatory response, leukocyte aggregation, and lipid peroxidation in the brain [205]. An acidic polysaccharide and a protein with the molecular weight (MW) of 14 kDa from *A. vera* have also been shown to have anti-inflammatory activity [2]. A cinnamic acid ester of aloesin found in *A. vera* has the ability to reduce croton oil-induced inflammation [148]. The C-glucosyl chromone isolated from *A. vera* gel extracts was also found to have anti-inflammatory properties [206]. The anthraquinones and chromones that are present in aloe inner gel possess strong anti-inflammatory effects in murine macrophages [200]. Fresh *A. vera* gel was reported for the significant reduction of acute inflammation in rats [207]. It is also reported that *A. vera* extract helped in decreasing inflammation by 48% in a rat arthritic inflammatory model [208].

### 7.4. Hepatoprotective Activity

Morphofunctional and molecular changes induced by carbon tetrachloride (CCl_4_) were reduced through aloe-emodin administration in rats [203]. Anthraquinone is likely to protect against hepatocyte death, lipid peroxidation, and the subsequent inflammatory response [133]. Phytosterols found in aloe, specifically lophenol and cycloartanol, have the ability to induce fatty acid synthesis downregulation and fatty acid oxidation upregulation in the liver, resulting in intra-abdominal fat reduction and hyperlipidemia improvement. An improvement in metabolic syndrome-related disorders and liver steatosis was obtained in aloe sterol-treated diabetic fatty rats. The results also showed that aloe suppressed obesity-induced inflammatory response by reducing cytokine levels [74]. *A. vera* gel extract can also prevent ethanol-induced fatty liver by suppressing mRNA lipogenic gene expression in the liver. *Aloe* gel also has the therapeutic potential to decrease cholesterol levels and cardiovascular disease risk [209]. In another study investigating *A. vera* extract effect on lindane (LD)-induced hepatoxicity and genotoxicity, leaf extract (1.0 mL/kg body weight, b.w.) decreased serum glutamate pyruvate transaminase (GPT), glutamate oxaloacetate transaminase (GOT), gamma-glutamyl transferase (GGT), and alkaline phosphatase (ALP) levels induced by 100 mg/kg b.w. LD [210]. In addition to *A. vera*, hepatoprotective activity was also reported for other *Aloe* genus species, including *A. arborescens*. *A. arborescens* was reported to be most active in liver diseases treatment, particularly cancers [211].

### 7.5. Antioxidant Effect

Free radical overproduction, including reactive oxygen species (ROS) resulting in oxidative stress, is known to be associated with the development of many diseases [226,243,277]. An antioxidant is a substance that significantly delays or inhibits oxidizable substrate oxidation at low concentrations [278]. In the literature, *A. vera* compounds were highlighted for their antioxidant activities [37,279,280]. It has been reported that *A. vera* leaf epidermis and flower methanol extracts exerted in vitro antioxidant effects [281]. *Aloe* gel showed free radical scavenging activity on 2,2-diphenyl-1-picrylhydrazyl (DPPH), 2,20-azinobis-(3-ethylbenzothiazoline-6-sulfonic acid) (ABTS)+•, and nitric oxide radicals [212]. *A. ferox* antioxidant capacity was determined using oxygen radical absorbance capacity (ORAC) and ferric reducing antioxidant power (FRAP) analyses. *A. ferox* antioxidant activity was attributed to its phytochemical composition. Thus, it can be used in alleviating symptoms or preventing oxidative stress-related diseases [44]. Aloeresins in *A. ferox* displayed strong antioxidant activity [213]. These 7-hydroxychromones, such as aloesin from an aloe extract, suppress free radical generation and reactive oxygen species (ROS) production, thereby preventing and treating ROS-mediated and other oxidative process-associated conditions [214]. Also, *A. ferox* leaves methanol extract showed good DPPH scavenging activity [215]. 5-Methylchromones aloesin, aloeresin A, and aloesone, which are compounds present in *A. barbadensis* and *A. arborescens*, exhibited the most radical scavenging activity by DPPH and ORAC assays [50]. The in vitro antioxidant activity of *A. arborescens* [94], *A. ferox* [44,58,59], *Aloe greatheadii* var. *davyana* [282], *A. harlana* [283], *A. saponaria* [284], *A. marlothii*, and *A. melanacantha* [285] leaf extracts were reported in the literature. Sazhina et al. [216] reported that leaf extracts from 15 *Aloe* species exhibited high antioxidant activity. However, the potent antioxidant activity measured in these in vitro studies needs to be followed up with appropriate in vivo assays. The absorption and bioavailability issues of such antioxidants following consumption also require investigation [198]. A protective potential of *Aloe* polysaccharides against 2,20-azobis(2-amidinopropane) dihydrochloride induced oxidative stress and cell death in kidney epithelial cells (Vero cells), as well as in an in vivo zebrafish model were shown [217]. Antioxidant compounds present in *A. saponaria* gel exerted antinociceptive and anti-inflammatory effects by the topical treatment of an ultraviolet B-induced sunburn model [286]. In another study, *A. barbadensis* extract displayed significant antioxidant activity in diabetic rats by increasing superoxide dismutase (SOD) enzyme activity and reducing lipid peroxidation [218].

### 7.6. Antibacterial, Antifungal, and Antiviral Activities

*A. vera* has in vitro antibacterial activity against Gram-negative and Gram-positive bacteria. Thus, it has been described as an antibacterial agent, as previously introduced. Antibacterial activity has been attributed to their polysaccharides, which trigger phagocytic leucocytes to destroy bacteria [75]. Indeed, *A. vera* gel extract antibacterial activity against Gram-positive and Gram-negative bacteria was showed in other studies [136,219]. An experiment proved that *A. vera* acetone extract was more active against *S. aureus*, *Streptococcus pyogenes*, *P. aeruginosa*, and *E. coli* compared to aqueous or ethanol extracts [6]. *A. ferox* is used to treat distinct infections, particularly those that are sexually transmitted, internal parasites, gonorrhoea, and syphilis in South Africa [220]. Aloe-emodin and aloin A isolated from *A. ferox* exhibited antibacterial activity against *Bacillus cereus*, *B. subtilis*, *S. aureus*, *S. epidermidis*, *E. coli*, and *Shigella sonnei*, while chrysophanol only led to *B. subtilis*, *S. epidermidis*, and *E. coli* growth inhibition [56]. Aloe-emodin and aloin A showed antibacterial activity against *B. subtilis*, *E. coli*, *S. epidermidis*, and *S. sonnei* [79]. An *Aloe* methanol extract and aloin had inhibitory effects against *Neisseria gonorrhoeae* [221]. The antimicrobial activities of 10 different South African *Aloe* species extracts were listed in another study [198]. Antimicrobial activities were reported for *A. arborescens* leaf ethyl acetate (against *E. coli*) [222], *A. barberae* root and leaf dichloromethane (against *E. coli* and *C. albicans*) [71], and *A. marlothii* leaf dichloromethane (against *S. aureus*) [223] extracts. Of particular interest are the inhibitory effects demonstrated by ethyl acetate and the methanol extracts of *A. arborescens* and *A. striatula* leaves, respectively, against ampicillin-resistant *E. coli* [222]. Pyrocatechol, a hydroxylated phenol that is present in *A. vera*, is known to have toxic effects on microorganisms [57,224]. The water and carbohydrates present in *A. vera* gel have very strong antibacterial potential against *P. aeruginosa* and mycobacterium strains, such as *Mycobacterium smegmatis*, *Mycobacterium fortuitum*, *Mycobacterium kansasii*, and *Mycobacterium tuberculosis* [192,225,226].

Aloe-emodin exerted *H. pylori* inhibition, which is the microorganism that is responsible for gastritis, peptic ulcer, gastric adenocarcinoma, and MALT (mucosa-associated lymphoid tissue) lymphoma [145]. In another study, *A. vera* gel exhibited inhibitory effects on multi-resistant *H. pylori* strains, with its activity being attributed to the anti-adhesive effect of gel polysaccharides [146]. Also, it was reported that *A. vera* inner gel has antibacterial activity against both susceptible and resistant *H. pylori* strains and it can be used as a natural agent for *H. pylori* gastric infection treatment [227].

The topical antibacterial as well as anti-inflammatory properties of aloe are embodied in a patent application of a laxative suppository preparation that is used for the treatment of hemorrhoids and bacterial infections of the anus [141].

A protein (MW 14 kDa) isolated from *A. vera* displayed antifungal activity against different *Candida* species, specifically *Candida parapsilosis*, *Candida krusei*, and *Candida albicans* [148]. *A. vera* removed *Candida* infections through promoting alkalization and alleviating constipation [228]. The saponins that are available strongly act against bacteria, viruses, fungi, and yeasts [67]. A report showed that *A.vera* pulp inhibitory activity on *Fusarium oxysporum* and its liquid fraction reduced *Rhizoctonia solani*, *F. oxysporum*, and *Colletotrichum coccodes* colony growth rates [229]. Some studies reported unspecified *A. ferox* ‘juice’ antifungal activity against *Trichophyton* spp. causing athlete’s foot and thrush [230]. Low activity was recorded for methanol extract against *C. albicans* [221]. Again, *A. ferox* acetone extract was found to be fungicidal against five fungi [170].

In many research studies, it has been reported that *A. vera* showed antiviral activity preventing virus adsorption, attachment, or entry into host cells. Zandi and Rastian [287] showed that *A. vera* gel had antiviral activity against herpes simplex virus (HSV) type 2 strains. Anthraquinone derivatives, such as aloe-emodin, emodin, and chrysophanol, which are present in aloe, have been reported to exhibit antiviral activity, also displaying inhibitory mechanisms against influenza A virus replication and virus-induced cytopathic effect [231]. *Aloe* was shown to increase CD_4_ count, which results from an immune system improvement. This may be beneficial to HIV-infected patients. It is thought that CD_4_ count raise can be attributed to in vitro HIV inhibition by acemannan or the immune-modulatory effects of aloe components [232]. Similarly, aloe-emodin displayed promising effects in reducing herpes simplex virus Type I and Type II infection. It can also inhibit viruses, such as *Varicella zoster* virus (VSV), influenza virus, and pseudorabies virus [181,233]. In various studies, it was reported that polysaccharide acemannan prevented human lung epithelial cell-bacteria adhesion, and also reduced herpes simplex virus infection [288,289,290]. In vitro and in vivo antiviral effects have also been observed for *A. ferox* and *A. secundiflora* leaf extracts, respectively [291,292].

### 7.7. Antiplasmodial/Antimalarial Activity

Malaria is a deadly disease that infects over 150 million humans annually in Africa alone [293]. The protozoan *Plasmodium falciparum* is the deadliest causative *Plasmodium* species. Traditionally, aloes are not known to possess antimalarial properties, but several scientific studies indicated that some *Aloe* species can be used to treat malaria-related symptoms. Van Zyl and Viljoen [294] evaluated 34 *Aloe* species and their main constituents for antiplasmodial activity using titrated [3*H*]-hypoxanthine incorporation assay. It was found that several methanol extracts inhibited *Plasmodium falciparum* growth by 50% in concentrations of 32–77 mg/mL. Clarkson et al. [295] in vitro tested 134 plant species against *P. falciparum* strain D10 using parasite lactate dehydrogenase (pLDH) assay. *A. ferox* organic extract (DCM, dichloromethane/MeOH 1:1) displayed promising antiplasmodial effects (IC_50_ 8 mg/mL), while aqueous extracts did not show any activity [295]. *Aloe marlothii* (whole plant) dichloromethane extract demonstrated good activity (IC_50_ of 3.5 mg/mL) against *P. falciparum* [295]. *A. maculata* [295] whole plant and *A. viridiflora* [294] leaf extracts showed mild activity. *A. marlothii* [295] root and *A. speciosa*, *A. suprafoliata*, and *A. wickensii* [294] leaf extracts showed weak activity. These findings indicate that the *Aloe* genus may be used as a potential antimalarial drug.

### 7.8. Anthelmintic Activity

The in vitro anthelmintic activity of the crude aqueous extract obtained from *A. ferox* was examined on eggs and larvae of the nematode parasite *Haemonchus contortus*. *A. ferox* extracts showed 100% egg hatch inhibition at 20 mg/mL and larval development inhibition at 2.5 mg/mL [296].

### 7.9. Anticancer Activity

Aloin, an anthraquinone that is a natural compound and the main *Aloe* ingredient, has been proposed as a potential therapeutic option in cancer, wherein it showed chemoprotective effects against 1,2-dimethylhydrazine-induced colon preneoplastic lesions in Wistar rats [234]. Indeed, aloin treatment inhibited vascular endothelial growth factor (VEGF) secretion in cancer cells. VEGF is one of the most important proangiogenic cytokines, which is known and well characterized as a tumor neovascularization inducer. Aloin treatment has shown to significantly inhibit VEGF-induced angiogenic response in human endothelial cells, in vitro, triggering proliferation inhibition and endothelial cell migration. Aloin was found to inhibit tumor angiogenesis and growth by signal transducer and activator of transcription 3 (STAT3) activation [235].

Aloe-emodin (1,8-dihydroxy-3-hydroxymethyl-9,10-anthracenedione) is an anthracenedione derivative from *A. vera* leaves. Recent reports have shown that aloe-emodin possesses antiproliferation effects in some cancer cells types, such as lung, squamous, glioma, and neuroectodermal cancer cells [236,237]. Aloe-emodin is also an anthraquinone subtype; it is a natural compound that has traditionally been found to have diverse biological activities, with anticancer functions among them [238,239]. Aloe-emodin has been found to possess an antiproliferation effect on some cancer cells types, inhibiting both N-acetyl transferase activity and gene expression. This effect plays a crucial role in aryl amine carcinogens metabolism, which is found in human malignant melanoma cells [240,241]. Recently, Lin et al. [241] demonstrated that aloe-emodin induced apoptosis in T24 human bladder cancer cells. Aloin, which is derived from *A. vera* leaves, has been shown to possess anticancer effects too [241], as it inhibits tumor angiogenesis and growth via blocking STAT3 activation, therefore displaying a potential as drug candidate for cancer therapy [242]. Anthraquinone derivatives, such as emodin-like natural (emodin, rhein, and aloin) and synthetic (anthraquinone-2-sulfonic acid) anthraquinones have recently been shown to protect amyloid β and aggregation-induced cell death through antiaggregating effects, and/or enhancing phosphatidylinositol-3-kinase/protein kinase B mediated survival mechanisms, which suggests that anthraquinone-2-sulfonic acid could be a new neuroprotective compound and a novel caspase inhibitor [148,243].

An investigation showed that polysaccharide acemannan inhibits benzopyrene binding with primary rat hepatocytes and thereby prevents cancer initiating benzopyrene DNA adduct formation [184,244]. In chemoprevention, *A. vera* gel stimulate glutathione S-transferase induction, which inhibits phorbol myristate acetate tumor-promoting effects [245,246]. Similarly, aloin inhibited cancer cells by altering a cell cycle through the mitochondrial-dependent pathway, which leads to cell membrane integrity loss and apoptosis [247]. A study demonstrated aloin protective effects on inducible nitric oxide synthase (iNOS) and NFκB synthesis induced in HaCat cells by ultraviolet (UV) B irradiation. Aloin inhibited NFκB and P65 activity by downregulating iNOS mRNA expression caused by UVB irradiation [248]. Aloe-emodin produced antitumor effects in P-glycoprotein overexpressing cell lines [249]. In addition, barbaloin, physcion, chrysophanol, aloesin, diethylhexyl phthalate, and an N-terminal octapeptide were reported to have cytotoxic activity on cancer cells [225,250,251,252,253]. Acemannan acts as a very active anticancer agent. Acemannan stimulated TNF-α, IL-1, and interferon production by macrophages and deactivated cancer cells [254]. In an experiment, aloin was tested on human uterine carcinoma HeLaS3 cells [297]. Aloin showed antiproliferative effects through arresting the cell cycle in the S phase and significantly increasing HeLaS3 cells apoptosis. This emphasized that aloin can be used for treating human cervical carcinoma in the future. It was also applied to radiosensitize HeLaS3 cells, which suggests an aloin cytotoxic adjuvant effect [298].

*A. ferox* is also used as an anticancer agent [299,300,301]. Aloe-emodin has been reported to have selective activity against neuroectodermal tumors, with practically no effect on normal cells [300]. Aloe-emodin promoted cell death through specific drug uptake by neuroectodermal tumors [302].

### 7.10. Antidiabetic Activity

The treatment of diabetes using various natural active compounds is still high-priority research. There are instances to consider *A. vera* as an antidiabetic agent. In vivo and in vitro studies strongly demonstrated that the water soluble fraction of *Aloe* species possesses glucose-lowering activities, and some of its components modulate glucose transporter-4 mRNA expression [255]. Studies have proved that the polysaccharides that are present in the plant protects β-cells from oxidative damage by alloxan [37,148,256]. It was reported that they helped reduce fasting blood glucose levels in alloxan-induced diabetic mice [257]. Polysaccharides play a major role in antidiabetic activities by increasing insulin levels, and hence, show hypoglycemic effects [183,258]. Phytosterols, such as lophenol, 24-methyl-lophenol, 24-ethyl-lophenol, cycloartanol, and 24-methylene cycloartanol were reported for their beneficial effects in diabetes and obesity [74]. A report gave confirmation of the clinical and experimental hypoglycemic conditions, due to *A. vera* sap oral intake continuously for four to 14 weeks [257]. One study discussed aloe-emodin-8-*O*-glycoside efficacy, isolated from *A. vera* gel, in enhancing glucose transport through proximal and distal marker modulation involved in glucose uptake and its transformation into glycogen [259]. Tanaka et al. [73] reported reductions in both the fasting and random blood glucose levels of db/db diabetic mice that were chronically treated with the same *A. vera* gel phytosterols [73]. Jain et al. [260] found that *A. vera* gel has significant antidiabetic and cardioprotective effects, as it significantly reduced oxidative stress in streptozotocin-induced diabetic rats and improved antioxidant status [260].

### 7.11. Antihyperlipidemic Activity

A recent study confirmed that phytosterols administration isolated from *A. vera* gel reduced visceral fat mass and improved hyperglycemia in Zucker diabetic fatty rats [261]. *Aloe succotrina* leaf dried pulp exerted remarkable antihyperlipidemic effects in high-fat diet and fructose-induced hyperlipidemic rats. It also significantly decreased the total serum cholesterol, total triglycerides, low-density lipoprotein (LDL), very low-density lipoprotein (VLDL), and high-density lipoprotein (HDL) cholesterol levels [262]. In polycystic ovarian syndrome (PCOS), rats treated with *A. vera* gel, plasma triglyceride, and LDL cholesterol levels decreased, while HDL cholesterol levels increased and PCOS were significantly reduced. *Aloe* gel also improved the reversion of abnormal estrous cyclicity, glucose intolerance, and lipid metabolizing enzyme activities [263,264,265].

### 7.12. Effect on Estrogen Status

Emodin and aloe-emodin isolated from *A. vera* gel suppressed breast cancer cell proliferation by targeting estrogen receptor-α protein stability through distinct mechanisms. This aspect suggests a possible anthraquinone application in preventing breast cancer cell proliferation through estrogen receptor-α inhibition [52]. *A. vera* gel also helped in maintaining ovarian steroid status in polycystic ovary-like condition, where steroidogenesis is altered and the estrogen and testosterone ratio is disturbed [263].

### 7.13. Antiulcer Activity

*A. vera* gel has the ability to minimize gastric ulcers in both humans and animals [30,267]. *A. vera* leaf extracts have also been widely recommended for digestion promotion and in peptic ulcer treatment due to its prominent cytoprotective action, whereby *A. vera* gel exhibited antibacterial activity against both susceptible and resistant *H. pylori* strains and acted as a promising effective natural agent in combination with antibiotics on *H. pylori* gastric infection treatment [5,266]. However, studies have confirmed that *A. vera* gel could not prevent ethanol-induced gastric lesions in rats [267]. In rats, gastric acid secretion and hydrochloric acid induced-gastric mucosa damage was investigated after *A. vera* aqueous ethanol extract application. *A. vera* extract exhibited dose-dependent inhibitory effects on gastric acid secretions by direct interaction with acid-producing cells or with H2-receptors on parietal cells. A report confirmed that *A. vera* extract showed cytoprotective activity at low doses [268]. Another report demonstrated that a mixed treatment with *A. vera* and sucralfate reduced gastric inflammation, enhanced epithelial cell proliferation, elongated gastric glands, and reduced ulcer sizes [269].

### 7.14. Treatment of Cardiovascular Disorders

*A. vera* can be used on cardiovascular disorders treatment. It stimulated fibroblast cells for making new tissues. Proteoglycans and collagens are formed, thus reducing cardiovascular disorders risk, after fibroblasts stimulation [190].

### 7.15. Skin Use

Collagen, elastin, and hyaluronic acid are the major components of the skin dermis layer. Collagen forms the three-dimensional structure of skin, and elastin maintains its elasticity, whereas hyaluronic acid restores skin moisture levels. Fibroblasts are mainly responsible for collagen activation, hyaluronic acid, and elastin in the skin dermis layer, and thereby maintain extracellular matrix homeostasis [270,271,272]. *Aloe* sterols promoted collagen production and thereby increased type I and type III collagen synthesis gene expression in human dermal fibroblasts. Sterols also increased the hyaluronic acid content of the dermal extracellular matrix. Therefore, they can improve skin moisture [273]. The melanocytic effects of *A. vera* leaf extract and aloin have been reported by Ali et al. [274], suggesting that they can be useful for hyperpigmentation treatment. Aloesin was reported to have tyrosinase inhibitory activity, which may be helpful in hyperpigmentation treatment, corresponding to melanin formation, such as melasma and ephelides [275]. Also, the skin cell proliferating activity of an *Aloe* glycopeptide (G1G1M1DI2) (MW 5.500 Da) was reported [303].

### 7.16. Anti-Aging Effect

*A. vera* was reported to trigger collagen and elastin fiber production, making skin more elastic and less wrinkled, but the exact mechanism is not well understood [4,177].

### 7.17. Antiallergic Activity

An *Aloe* glycoprotein (10 kDa) was found to reduce histamine release and promote leukotriene synthesis and secretion in the activated lung mast cells of guinea pigs. Furthermore, glycoprotein dose-dependently decreased protein kinase C and phospholipase C activities, inhibiting diacylglycerol and phospholipase A activity and blocking Ca^2+^ influx during mast cell activation [276].

### 7.18. Effect on Central and Peripheral Nervous Systems

Following *Aloe* extracts administration, marked improvements in learning, memory, and cognitive function and Alzheimer disease have also been reported [304,305,306,307]. *Aloe* gel potential on Parkinson disease has also been reported in mice [308]. Moreover, *Aloe* leaf powder aqueous extract showed anticonvulsant activity in mice [309].

## 8. Clinical Efficacy of *Aloe* Plants in Humans

*Aloe* is known as healing plant. It has been used for traditional medical purposes in several cultures [220], and its distinct therapeutic properties have been reported (Table 7). Some of them are attributed to the specific compounds that are present in *Aloe*.

### 8.1. Wound Healing and Cell Proliferation

In traditional medicine, *A. ferox* leaves and roots are applied topically, sometimes mixed with animal fat, or taken internally to treat eczema, dermatitis, and acne. They are also used in the treatment of various other skin diseases or conditions, such as skin cancer, burns, and psoriasis [44]. Epidermal melanin overproduction, synthesized by tyrosinase action, causes skin hyperpigmentation. The aloesin and arbutin present in *Aloe* can inhibit tyrosinase activity in a synergistic manner [310]. In fact, it has been reported that aloesin can inhibit hyperpigmentation in human skin after UV radiation in a dose-dependent manner, while co-treatment with aloesin and arbutin exerted an additive effect [311]. *A. barbadensis* [312] and *A. arborescens* [313] wound-healing properties were also reported. In a clinical study, burn wound healing was found to be remarkably earlier in *A. vera*-treated patients than in those who were treated with 1% silver sulfadiazine cream and a burn dressing for superficial and partial thickness burn treatment [314]. In another clinical study, it was shown that re-epithelialization and partial thickness burn wound healing was significantly faster in *Aloe-*treated sites compared to the silver sulfadiazine-treated ones. The results confirmed that *Aloe* cream can be used to treat second-degree burn wounds, too [315]. A significant improvement in burning wound healing after *A. vera* treatment was observed in rat models [316,317].

### 8.2. Anti-Inflammatory and Immunomodulatory Effects

A report of a clinical study has shown that the oral administration of 2% *A. vera* gel is not only effective in decreasing pain score and wound size in recurrent aphthous stomatitis patients, but also in decreasing the aphthous wound-healing period [266]. It has been reported that *A. vera* extracts may be used to treat the external parts of eye inflammations, such as the cornea [318]. *A. vera* gel had strong immunomodulatory activity, downregulating lipopolysaccharide-induced inflammatory cytokine production and NLRP3 (NACHT, LRR, and PYD domains-containing protein 3) inflammasome expression in human macrophages [319]. Acemannan stimulated immunity through potentiating the lymphocyte response to alloantigen with nitric oxide production activation by macrophages and cytokines, such as interleukin (IL)-1, IL-6, interferon (IFN), and TNF-α. It enhanced phagocytosis and also increased circulating monocyte and macrophage levels [254,320,321]. Polysaccharide aloeride activated nuclear factor (NF)-ĸB in human macrophages similar to bacterial endotoxin [75].

### 8.3. Antidiabetic Activity

In general, the α-amylase that is present in human pancreatic cell helps control starch hydrolysis inside the body, and hence protects from postprandial hyperglycemia. Postprandial hyperglycemia is characterized by a rapid increase in blood glucose levels in diabetic patients. An investigation confirmed that *A. vera* decreased diabetes severity by lowering blood glucose levels in type 2 diabetic patients [322]. Other clinical studies have suggested that *A. vera* gel may act as a safe antihyperglycemic and antihypercholesterolemic agent in type 2 diabetic patients, without any significant effects on other normal blood lipid levels or liver/kidney function [323].

In a randomized controlled trial, *A. vera* gel complex reduced body weight, body fat mass, and insulin resistance in obese pre-diabetic and early non-treated diabetic patients [322]. Further, in a pilot study, two *Aloe* products in pre-diabetic patients over an eight-week period, tended to revert impaired fasting glucose levels and impaired glucose tolerance observed in pre-diabetes/metabolic syndrome conditions [322].

### 8.4. Antihyperlipidemic Activity

*A. vera* antihyperlipidemic activity has also been reported. It had beneficial effects on fatty streak development prevention, and may help in reducing atherosclerosis development through risk factor modification [323]. *A. vera* leaf gel efficacy was checked in hyperlipidemic type 2 diabetic patients in a randomized double-blind placebo-controlled clinical trial, wherein it reduced total cholesterol and low-density lipoprotein (LDL) levels [323].

### 8.5. Treatment of Acquired Immune Deficiency Syndrome (AIDS)

It has been shown that acquired immune deficiency syndrome (AIDS) can be treated by using *A. vera* extracts in many reports. A daily intake of a minimum dose of 1200 mg of *A. vera* active ingredients improved AIDS symptoms. It soothed the wound and burn of internal organs, and hence acted as a promising AIDS drug. The mannose-6-phosphate present in *A. vera* can also inhibit the HIV-1 virus that causes AIDS [324].

### 8.6. Effect on Dental and Oral Diseases

It has been shown that acemannan hydrogels heal aphthous ulcers and reduce pain [325]. *A. vera* is very effective for gum diseases, such as gingivitis and periodontitis [327]. Besides, it has been reported that *A. vera* mouthwash reduced plaque and gingivitis, though less than chlorhexidine [326].

## 9. Conclusions and Future Perspectives

The therapeutic effects of *Aloe* species in traditional and modern medicines are well documented. The present report highlights the research progress on *Aloe* spp. botany, phytochemical composition, ethnobotanical uses, food preservation, and preclinical and clinical efficacy. Of special attention are aloin A, aloin B, aloinoside A, aloinoside B, aloesin, plicataloside, isovitexin, and aloe-emodin, because of their prominent biological activity and abundance in *Aloe* plants. Therefore, considering the data presented here, and namely taking into consideration both its valuable phytoconstituents and wide beneficial effects, the *Aloe* species may be considered as economically important matrices for food, medical, and pharmaceutical industries.

## Figures and Tables

**Table 1:** Phytoconstituents of *Aloe* species detected by high-performance liquid chromatography (HPLC) and reversed-phase HPLC.

*Aloe* Species	Phytochemicals	Reference
**Leaves**		
*A. africana*	Aloesin D, aloin A, aloin B, aloinoside A, and aloinoside B	[33]
*A. arborescens* ^1^	Aloenin, a phenyl pyrone, aloesin, aloeresin, aloin B, aloin A	[34]
	Aloe emodin-diglucoside, lucenion II, 6′-*O*-caffeoyl-5-hydroxyaloin A, vicenin II, *trans*-*p*-coumaric derivatives, 3-*O*-(*E*)-caffeoyl-4-*O*-feruloylquinic acid, luteolin-*O*-xylosylglucoside malonylated, aloeresin C isomer, isorhamnetin-3-*O*-deoxyhexosyl(1-6) hexoside, 7-*O*-methyl kaempferol dimmer, caffeoyl quinic acid hexoside, kaempferol-3-*O*-hexosyl-*O*-pentoside, orientin, 3-*O*-caffeoyl-5-*O*-coumaroylquinic acid, 4-succinyl-3,4-dicaffeoylquinic acid, 6′-malonylnataloin, cholestenol, isoquercetrin, aloinoside A/B, 3,4-di-*O*-(*E*)-*p*-coumaroylquinic acid, 2′-*O*-feruloylaloesin, 7-*O*-methylaloesin-penta acetate, malonyl-4,5-*O*-dicaffeoylquinic acid, nataloin, veracylglucan A, aloenin B, wighteone-*O*-diglucoside malonylate, aloin A, caffeoylester of aloesin, aloeresin E, barbaloin (10R)/isobarbaloin (10S), quercetin-7-*O*-hexoside-3-*O*-malonylhexoside, aloe-emodin-8-*O*-glucoside, chrysophanol-8-*O*-(6′-*O*-galloyl-) glucoside, aloeresin H, and pentahydroxyflavonol-*O*-hexosyl rhamnoside	[35]
*A. archeri*	Plicataloside	[36]
*A. babatiensis*	Plicataloside	[36]
*A. barbadensis*	8-*C*-*b*-d-[2-*O*-(*E*)-coumaroyl]glucopyranosyl[2-[2-hydroxy]propyl-7-methoxy-5-methylchromone, aloeresin D, *C*-glucosyl chromone, and alcohol	[37]
*A. boscawenii*	Aloin A, aloin B, aloinoside A, aloinoside B, microdontin A, and microdontin B	[33]
*A. brachystachys* ^1^	Aloesin, aloenin, aloin B, and aloin A	[34]
*A. brandhamii* ^1^	Aloesin, aloenin, aloin B, and aloin A	[34]
*A. brevifolia*	*cis*-*p*-Coumaric acid derivatives, 5-*O*-caffeolyquinic acid, vicenin II, luteolin-*O*-xylosylglucoside malonylated, aloeresin C isomer, epi-catechin digalloyl rhamnoside, isorhamnetin-3-*O*-deoxyhexosyl(1-6) hexoside, caffeoyl quinic acid hexoside, 4-succinyl-3,4-dicaffeoylquinic acid, nataloin, cholestenol, 2’-*O*-feruloylaloesin, isoaloeresin D, and aloeresin	[35]
*A. brunneostriata*	Dihydroisocoumaringlucoside, aloin A, aloin B, aloinoside A, aloinoside B, microdontin A, and microdontin B	[33]
*A. buchlohii*	Aloin A, aloin B, microdontin A, and microdontin B	[33]
*A. bussei* ^1^	aloesin, aloenin, aloin B, and aloin A	[34]
*A. calidophila*	Dihydroisocoumaringlucoside, aloin A, aloin B, aloinoside A, aloinoside B, microdontin A, and microdontin B	[33]
*A. cameronii*	Aloesin, aloeresin A, aloeresin D, aloin A, aloin B, aloinoside A, aloinoside B, microdontin A, and microdontin B	[33]
*A. camperi*	Dihydroisocoumaringlucoside, aloin A, aloin B, aloinoside A, aloinoside B, microdontin A, and microdontin B	[33]
*A. canarina*	Aloesin, aloeresin D, aloin A, aloin B, aloinoside A, aloinoside B, microdontin A, and microdontin B	[33]
*A. chabaudii*	Plicataloside	[36]
*A. cheranganiensis* ^1^	Aloesin, 7-*O*-methylaloesin, aloenin, aloeresin A, aloeresin D, aloin B, aloin A	[34]
*A. chrysostachys*	7-*O*-Methylaloesin, aloeresin D, aloin A, aloin B, aloinoside A, aloinoside B, microdontin A, and microdontin B	[33]
*A. classenii* ^1^	Aloesin, aloenin, aloeresin D, aloin B, and aloin A	[34]
*A. dawei* ^1^	Aloesin, aloenin, aloeresin D, aloin B, and aloin A	[34]
*A. deserti*	Plicataloside	[36]
*A. diolii*	Aloin A, aloin B, aloinoside A, aloinoside B, microdontin A, and microdontin B	[33]
*A. dorotheae* ^1^	Aloesin, aloenin, aloin B, and aloin A	[34]
*A. elegans*	Dihydroisocoumaringlucoside, aloin A, aloin B, aloinoside A, aloinoside B, microdontin A, and microdontin B	[33]
*A. eru*	Vicenin II, 3-*O*-(*E*)-caffeoyl-4-*O*-feruloylquinic acid, iso pentyldihexose, apigenin-7-*O*-glycuronyl, aloenin, nataloin, cholestenol, isoquercetrin, aloinoside A/B, epi (afzelechin)—(epi) gallocatechin, 2′-*O*-feruloylaloesin, 7-methylether of 2′-feruloylaloesin, glucuronides, isoaloeresin D, aloeresin, kaempferol di deoxyhexosylhexoside, aloenin B, caffeoylester of aloesin, aloeresin E, apigenin-7-*O*-glycuronyl, aloe-emodin-8-*O*-glucoside, and 2′-*p*-methoxycoumaroylaloresin	[35]
*A. ferox*	*cis*-*p*-Coumaric acid derivatives, aloe emodin-diglucoside, lucenin II, vicenin II, orientin, 6’-malonylnataloin, kaempferol di deoxyhexosylhexoside, aloeresin E, quercetin pentosyl rutinoside, aloe-emodin-8-*O*-glucoside, and chrysophanol-8-*O*-(6′-*O*-galloyl-) glucoside	[35]
	Aloin (A and B), aloinoside (A and B) and microdontin (A and B), aloesin, and aloeresin A	[33]
*A. fibrosa*	Plicataloside	[36]
*A. fleurentiniorum*	Dihydroisocoumaringlucoside, aloin A, aloin B, aloinoside A, aloinoside B, microdontin A, and microdontin B	[33]
*A. flexilifolia*	Aloesin, 7-*O*-methylaloesin, aloeresin D, aloin A, aloin B, aloinoside A, aloinoside B, microdontin A, and microdontin B	[33]
*A. francombei*	Plicataloside	[36]
*A. gilberti*	Aloesin, 7-*O*-methylaloesin, aloin A, aloin B, aloinoside A, aloinoside B, microdontin A, and microdontin B	[33]
*A. gossweileri* ^1^	Aloesin, aloenin, aloin B, and aloin A	[34]
*A. grandidentata*	Aloesin, aloe emodin-diglucoside, caffeoyl ferulic acid derivatives, chrysoeriol-7-*O*-glycuronyl, lucenin II, 6′-*O*-caffeoyl-5-hydroxyaloin A, vicenin II, 3-*O*-(*E*)-caffeoyl-4-*O*-feruloylquinic acid, luteolin-*O*-xylosylglucoside malonylated, isorhamnetin-3-*O*-deoxyhexosyl(1-6) hexoside, kaempferol-3-*O*-hexosyl-*O*-pentoside, orientin, isoorientin, 5-hydroxyaloin A, nataloin, cholestenol, aloinoside A/B, 3,4-di-*O*-(*E*)-*p*-coumaroylquinic acid, epi(afzelechin)-(epi)gallocatechin, 2′-*O*-feruloylaloesin, 7-methylether of 2′-feruloylaloesin, isoaloeresin D, aloeresin, nataloin, aloenin B, aloin A, aloin B, hydroxy octadecenic acid, trihydroxycinnamic acid derivatives, aloeresin E, acetyl dicaffeoylquinic acid, and kaempferol-3-*O*-malonylhexoside	[35]
*A. guillaumetii*	7-*O*-Methylaloesin, aloin A, aloin B, aloinoside A, and aloinoside B	[33]
*A. harlana*	Aloin A, aloin B, aloinoside A, aloinoside B, microdontin A, and microdontin B	[33]
*A. hemmingii*	Aloesin, 8-*O*-methyl-7-hydro-xyaloin, aloin A, aloin B, aloinoside A, and aloinoside B	[33]
*A. kedongensis* ^1^	Aloesin, 7-*O*-methylaloesin, aloenin, nataloin B, and nataloin A	[34]
*A. labworana*	Plicataloside	[36]
*A. leachii* ^1^	Aloesin, aloenin, aloeresin D, aloin B, and aloin A	[34]
*A. lensayuensis*	Aloin A, aloin B, aloinoside A, aloinoside B, microdontin A, and microdontin B	[33]
*A. leptosiphon* ^1^	Aloesin, aloenin, aloin B, and aloin A	[34]
*A. mcloughlinii*	Aloesin, dihydroisocoumaringlucoside, aloeresin D, aloin A, aloin B, aloinoside A, and aloinoside B	[33]
*A. megalacantha*	Dihydroisocoumaringlucoside, aloin A, aloin B, aloinoside A, aloinoside B, microdontin A, and microdontin B	[33]
*A. microdonta*	Aloin A, aloin B, aloinoside A, aloinoside B, microdontin A, and microdontin B	[33]
*A. monticola* ^1^	Aloesin, aloenin, aloeresin D, aloin B, and aloin A	[34]
*A. morijensis*	Plicataloside	[36]
*A. multicolor*	Plicataloside	[36]
*A. murina*	Plicataloside	[36]
*A. ngongensis*	Aloeresin D, aloin A, aloin B, aloinoside A, aloinoside B, microdontin A, and microdontin B	[33]
*A. nyeriensis* ^1^	Nataloe-emodin, nataloe-emodin-2-*O*-Glc, nataloin, aloenin, aloenin aglycone, and aloenin-2″-*p*-coumaroyl ester	[38]
*A. otallensis*	Plicataloside	[36]
*A. palmiformis*	Plicataloside	[36]
*A. parvidens*	Plicataloside	[36]
*A. peckii*	Aloesin, dihydroisocoumaringlucoside, aloeresin D, aloin A, aloin B, aloinoside A, and aloinoside B	[33]
*A. peglerae*	Aloesin, aloeresin E, aloeresin F, homonataloin A, and homonataloin B	[39]
*A. penduliflora*	7-*O*-Methylaloesin, dihydroisocoumaringlucoside, aloin A, aloin B, aloinoside A, and aloinoside B	[33]
*A. perfoliata*	Aloesin, 1-hexanol-pentosylhexoside, 3-*O*-(*E*)-caffeoyl-4-*O*-feruloylquinic acid, luteolin-*O*-xylosylglucoside malonylated, aloeresin C isomer, epi-catechin digalloyl rhamnoside, 7-*O*-methyl kaempferol dimmer, orientin, 3-*O*-caffeoyl-5-*O*-coumaroylquinic acid, 4-succinyl-3,4-dicaffeoylquinic acid, 5-hydroxyaloin A, aloinoside A/B, epi (afzelechin)—(epi)gallocatechin, 7-*O*-Mmethylaloesin-penta acetate, glucuronides, isoaloeresin D, isovitexin, 6′-*O*-coumaroyl aloesin, aloeresin A isomer, caffeoylester of aloesin, aloeresin E, acetyl dicaffeoylquinic acid, quercetin-7-*O*-hexoside-3-*O*-malonylhexoside, aloe-emodin-8-*O*-glucoside, 2′-*p*-methoxycoumaroylaloresin, aloeresin H, pentahydroxyflavonol-*O*-hexosyl rhamnoside, and kaempferol-3-*O*-malonylhexoside	[35]
*A. plicatilis*	Plicataloside	[36]
*A. pustuligemma*	Plicataloside	[36]
*A. rabaiensis*	Aloeresin D, aloin A, aloin B, aloinoside A, and aloinoside B	[33]
*A. rivae*	Dihydroisocoumaringlucoside, aloin A, aloin B, aloinoside A, aloinoside B, microdontin A, and microdontin B	[33]
*A. rugosifolia*	Plicataloside	[36]
*A. saponaria*	*cis*-*p*-Coumaric acid derivatives, 3,4-di-*O*-(*E*)-caffeoylquinic acid, malonyl-3,4-*O*-dicaffeoyl quinic acid, lucenin II, luteolin-*O*-xylosylglucoside malonylated, isorhamnetin-3-*O*-deoxyhexosyl(1-6) hexoside, 4-succinyl-3,4-dicaffeoylquinic acid, 2′-*O*-feruloylaloesin, 7-*O*-methylaloesin-penta acetate, 7-methylether of 2′-feruloylaloesin, trihydroxy octadecenoic acid, quercetin-7-*O*-hexoside-3-*O*-malonylhexoside, aloe-emodin-8-*O*-glucoside, 2′-*p*-methoxycoumaroyl-aloresin, aloeresin H, and tetra-*O*-methyl ether	[35]
*A. scabrifolia*	Dihydroisocoumaringlucoside, aloin A, aloin B, aloinoside A, aloinoside B, microdontin A, and microdontin B	[33]
*A. schelpei*	8-*O*-Methyl-7-hydroxyaloin, aloin A, aloin B, microdontin A, and microdontin B	[33]
*A. schweinfurthii*	Plicataloside	[36]
*A. scobinifolia*	Dihydroisocoumaringlucoside, aloin A, aloin B, aloinoside A, aloinoside B, microdontin A, and microdontin B	[33]
*A. secundiflora*	Aloenin, aloenin B, isobarbaloin (aloin B), barbaloin (aloin A), aloinside A, aloinside B, aloesin derivative, and an unidentified mixture of dimers	[40]
*A. secundi* *flora* ^1^	Aloenin, aloin B, and aloin A	[34]
*A. sinkatana*	8-*O*-Methyl-7-hydroxyaloin, aloin A, aloin B, aloinoside A, and aloinoside B	[33]
*A. somaliensis*	Dihydroisocoumaringlucoside, aloin A, aloin B, aloinoside A, aloinoside B, microdontin A, and microdontin B	[33]
*A. steudneri*	Dihydroisocoumaringlucoside, 8-*O*-methyl-7-hydroxyaloin, aloin A, aloin B, microdontin A, and microdontin B	[33]
*A. tewoldei*	Dihydroisocoumaringlucoside, aloin A, aloin B, aloinoside A, aloinoside B, microdontin A, and microdontin B	[33]
*A. tormentorii*	Phenols, saponins, tannins, alkaloids, anthraquinones, terpenes, coumarins and flavonoids	[41]
*A. tororoana* ^1^	7-*O*-Methylaloesin, aloenin, aloin B, and aloin A	[34]
*A. tugenensis*	Plicataloside	[36]
*A. tweediae*	Dihydroisocoumaringlucoside, aloeresin D, aloin A, aloin B, aloinoside A, and aloinoside B	[33]
*A. ukambensis*	Plicataloside	[36]
*A. vera*	*cis*-*p*-Coumaric acid derivatives, malonyl-3,4-*O*-dicaffeoyl quinic acid, lucenin II, 6′-*O*-caffeoyl-5-hydroxyaloin A, vicenin II, trans-*p*-coumaric derivatives, luteolin-*O*-xylosylglucoside malonylated, aloeresin C isomer, isorhamnetin-3-*O*-deoxyhexosyl(1-6) hexoside, 7-*O*-methyl kaempferol dimmer, caffeoyl quinic acid hexoside, orientin, isoorientin, 3-*O*-caffeoyl-5-*O*-coumaroylquinic acid, 6’-malonylnataloin, aloinoside A/B, 7-*O*-methylaloesin-penta acetate, malonyl-4,5-*O*-dicaffeoylquinic acid, nataloin, aloenin B, wighteone-*O*-diglucoside malonylate, aloin A, aloin B, aloeresin E, barbaloin (10R)/isobarbaloin (10S), quercetin-7-*O*-hexoside-3-*O*-malonylhexosidea, and aloe-emodin-8-*O*-glucoside	[35]
*A. wredfordii*	Plicataloside	[36]

^1^ Reversed-phase HPLC.

**Table 2:** Phytoconstituents of *Aloe* species detected by gas chromatography coupled to mass spectrometry (GC–MS).

*Aloe* Species	Phytochemicals	Reference
**Leaves**		
*A. albiflora*	Trimethylsilyl ether 2-hexanol, benzene acetaldehyde, lactic acid, benzyldimethylsilyl ester hydrocinnamic acid, 2,4-dimethyl benzaldehyde, 2-ethyl phenol, trimethylsilyl ether 9-decen-1-ol, trimethylsilyl ester benzene acetic acid, trimethylsilyl ester nonanoic acid, 2-methoxy-3-(2-propenyl)-phenol, 3-(2-trimethylsilyloxyethyl)-phenol, methyleugenol, phenyl 1,2-ethanediol, 2,4-bis(1,1-dimethylethyl)-phenol, 2-methyl-1-hexadecanol, nonadecane, 1-methylethyl ester dodecanoic acid, lauric acid, β-bisabolol, 2,6,10-trimethyl-tetradecane, tert-hexadecanethiol, tetradecanoic acid, pentadecanoic acid, ethyl ester cholestenol acid, trimethylsilyl ester *cis*-9-hexadecenic acid, palmitic acid, cis-13-eisosenoic acid, heptadecanoic acid, ethyl 9,12,15-octadecatrienoate, linoleic acid, α-linolenic acid, octadecanoic acid, eicosanoic acid, squalene, and β-sitosterol	[42]
*A. aristata*	Benzeneacetaldehyde, lactic acid, 2-methyl-2-indecanethiol, 2,5-dimethyl-benzaldehyde, 2-ethyl-phenol, trimethylsilyl ether 3-ethylphenol, *p*-ethylguaiacol, phosphoric acid, tridecane, (*E*)-3-eicosene, 2-methyl-1-hexadecanol, 2,6,11-trimethyl-dodecane, eugenol, vanillin, methyleugenol, hexadecane, 2,4-bis(1,1-dimethylethyl)-phenol, nonadecane, 1-methlethyl ester dodecanoic acid, lauric acid, 2,6,10-trimethyl tetradecane, 2,6,10,15-tetramethyl heptadecane, trimethylsilyl ester myristic acid, pentadecanoic acid, ethyl palmitate, trimethylsilyl ester *cis*-9-hexadecenoic acid, palmitic acid, trimethylsilyl ester *cis*-10-heptadecenoic acid, heptadecanoic acid, ethyl ester 9,12-octadecadienoic acid, ethyl 9,12,15-octadecatrienoate, linoleic acid, α-linolenic acid, octadecanoic acid, α-amyrin, squalene, ethyl iso-allocholate, and β-sitosterol	[42]
*A*. *aspera*	Trimethylsilyl ether 2-pentanol, m-pyrol, lactic acid, 3,5-dimethyl-benzaldehyde, benzoic acid, succinic acid, fumaric acid, nonanoic acid, tetradecane, trimethylsilyl ester decanoic acid, 2,4-bis(1,1-dimethylethyl)-phenol, 2,3,5,8-tetramethyl-decane, 2-methyl-1-hexadecanol, hexadecane, 1-methylethyl ester dodecanoic acid, lauric acid, nonadecane, 2,6,10,15-tetramethyl-heptadecane, 2,6,10-trimethyl-tetradecane, azelaic acid, isopropyl ester myristic acid, tetradecanoic acid pentadecanoic acid, trimethylsilyl ester cis-9-hexadecenoic acid, palmitic acid, heneicosane, heptadecanoic acid, linoleic acid, trimethylsilyl ester oleic acid, octadecanoic acid, eicosanoic acid, trimethylsilyl ester 1-docosanol, docosanoic acid, squalene, trimethylsilyl ester tetracosanoic acid, γ-tocopherol, ethyl iso-allocholate, 1-heptatriacotanol, campesterol, stigmasterol, β-sitosterol	[42]
*A. excelsa*	Limonene, carvone, and 2-phenylacetonitrile	[43]
*A. ferox*	Polyphenols/phenolic compounds (phenol, gentisic, cholestenol, homovanilic, *O*-hydroxycinnamic, protocatechuic, 3,4-dihydroxyphenylacetic, 5-methoxyprotocatechuic, syringic, sinapic, *p*-coumaric, caffeic, isoferulic, ferulic, 4-methoxycinnamic, aloe emodin, 4-phenyllactic, 4-ethylphenol, *p*-toluic, hydrocinnamic, *p*-salicylic, benzoic, mandelic, hydroxyphenylacetic, pyrocatechuic, hydro-*p*-coumaric, and 6,7-dihydroxycoumarin); organic acids (isovaleric, lactic, glycolic, furoic, 3-hydroxypropionic, 2-hydroxyvaleric, cyclohexanone-3-carboxylic, 3-hydroxyisovaleric, 3-methyl-1,3-hydroxybutanoic, 2-hydroxycaproic, 2-ketoisovaleric, succinic, 2-methylsuccinic, methylmalic, malic, 3,4,5-trihydroxypentanoic, d-ribonic, suberic, 3-hydroxypicolinic, and isonicotinic); fatty acids (lauric, myristic, cholestenol, palmitoleic, palmitic, stearic, linoleic, oleic, linolenic, erucic, cholestenol, arachidic, heneicosanoic, behenic, tricosanoic, lignoceric and pentacosanoic); alkaloids (hypoxanthine and xanthine); indoles (indole-5-acetic acid, and indole-3-acetic acid); pyrimidines (uracil and thymine); alkanes (1,3-dihydroxybutane); sterols (cholestenol, campestrol, â-sitosterol, and stigmasterol); dicarboxylic acids (azelaic and undecanedioic), and ketones (4,6-dimethyl-2-heptanone, acetophenone, and 2,4-dimethyl-4-heptanone)	[44]
*A. jucunda*	Benzaldehyde, lactic acid, 2-ethyl phenol, benzoic acid, ester octanoic acid, phenylacetic acid, 4-ethyl-1,2-benzene, dimethoxy-benzaldehyde, 4-vinylveratrole, eugenol, tetradecane, methyleugenol, 2-allyl-1,4-dimethoxy-3-methyl-benzene, nonadecane, 3,5-bis(1,1-dimethylethyl)-phenol, 1,2-dimethoxy-4-(2-methoxyethenyl)benzene, 1-dodecanol, estragole, dodecanoic acid, 1-methylethyl ester, 4-hydroxybenzoic acid lauric acid, β-bisabolol, 2,6,10-trimethyl-tetradecane, geranyl isovalerate, tetradecanoic acid, 3,5-bis(1,1-dimethylethyl)-4-benzoic acid, methyl ester hexadecenoic acid, 2,4,6-tris(1,1-dimethylethyl)-phenol, ethyl palmitate, trimethylsilyl ester palmitelaidic acid, palmitic acid, methyl ester linolenic acid, heptadecanoic acid, ethyl ester 9,12-octadecadienoic acid, ethyl 9,12,15-octadecatrienoate, ethyl ester stearic acid, linoleic acid, α-linolenic acid, octadecanoic acid, ester eicosanoic acid, bumetrizole, trimethylsilyl ester cis-13-docosenoic acid, ester docosanoic acid, squalene, heptacosane, ethyl iso-allocholate, and β-amyrin	[42]
*A. vera*	Debocane, 4-methyl, tricosane, 6-hydroxyhexane-3-1, 1-dodecanol, 1-octadecanol, cholestenol acid, 9-octadecenoic acid, octadecanoic acid, 1-(phenylthioxomethyl)piperidine, docosane, sitosterol, and stigmasterol	[45]
	Phenolic acids or polyphenols (phenol, cholestenic acid, homovanilic acid, protocatechuic acid, 3,4-dihydroxyphenylacetic acid, 5-methoxyprotocatechuic acid, and syringic acid. Sinapic acid, *p*-coumaric acid, isoferulic acid, ferulic acid, aloe emodin, 4-phenyllactic acid, 4-ethylphenol, hydrocinnamic acid, *p*-salicylic acid, benzoic acid, and hydro-*p*-coumaric acid); alcohols (2-butanol, glycerol, and phenylethanol); aldehydes (benzaldehyde and m-tolualdehyde); organic acids (lactic acid, glycolic acid, pyruvic acid, furoic acid, phosphoric acid, succinic aid, 2-methylsuccinic acid, malicnaicd, tartaric acid, and isonicotinic acid); alkanes (1,3-dihydroxybutane); pyrimidines (uracil and thymine) fatty acids (lauric acid, myristic acid, palmitoleic acid, and linoleic acid) indoles (indole-3-acetic acid); alkaloids (hypoxanthine); ketones (acetophenone); sterols (cholestenol and β-sitosterol); dicarboxylic acids (azelaic acid and undecanedioic acid)	[6]
	Ethylene glycol, propylene glycol, 2,3-bis(trimethylsiloxy)-butane, glycolic acid, 6-methyl-octadecane, 4-ethylbenzaldehyde, 4-hydroxybutyric acid, benzoic acid, (±)-2-Hydroxyoctanoic acid, octanoic acid, succinate, methyl succinic acid, glyceric acid, fumaric aicd, nonanoic acid, 2-methoxy-3-(2-prophenyl)-phenol, teradecane, methyleugenol, glutarate, 4-allyl-2-methoxyphenoxy-i-dodecanol, β-copaene, malic acid, adipic acid, 2-methyl-1-hexadecanol, bis(trimethylsilyl-pyroglutamic acid, m-hydroxybenzoic acid, pimelic acid, 1-methylethyl ester dodecanoic acid, 4-hydroxybenzoic acid, lauric acid, 1-[(trimethylsilyl)oxy]-2-methylanthraquinone, suberic acid, geranyl isovalerate, 2,6,10-timethyl tetradecane, bis(trimethylsilyl) ester 1,3-benzenedicarboxylic acid, azelaic acid, protocatechuic acid, tetradecanoic acid, sebacic acid, pentadecanoic acid, 2,4,6-tris(1,1-dimethylethyl phenol, undecadioic acid, trimethylsilyl ester palmitelaidic acid, palmitic acid, phytol, (2*E*)-3,7,11,15-tetramethyl-2-hexadecenyl trimethylsilyl ether, dodecanedioic acid, linoleic acid, α-linolenic acid, octadecanoic acid, eicosanoic acid, 2-monopalmitoylglycerol trimethylsilyl ester, 1-monopalmitin trimethylsilyl ester, docosanoic acid, 9-octadecenoic acid, 1,3-bis-(OTMS)-2-propyl ester, squalene, trimethylsilyl ester tetracosanoic acid, heptacosane, O-trimethylsilyl-(+)-α-tocopherol, β-sitosterol and β-amyrin	[42]

**Table 3:** Phytoconstituents of *Aloe* species detected by high-performance liquid chromatography and thin-layer chromatography (TLC).

*Aloe* Species	Phytochemicals	Reference
**Leaves**		
*A. bakeri*	Dihydroisorhamnetin	[46]
*A. bellatula*	Flavonoids	[46]
*A. boylei*	Isovitexin	[46]
*A. chortolirioides* var. *chortolirioides*	Aloesin, nataloin A and B, and 7-hydroxyaloin	[46]
*A. chortolirioides* var. *wooliana*	Isovitexin, aloesin, aloin A and B, and 7-hydroxyaloin	[46]
*A. christianii*	Homonataloin A and B, aloeresin A, and nataloin A and B (anthrones)	[46]
*A. ciliaris*	Isovitexin and aloeresin A	[46]
*A. commixta*	Isovitexin	[46]
*A. ecklonis*	Isovitexin	[46]
*A. glauca*	Isovitexin, trace of dihydroisorhamnetin, and aloesin (a type of chromone)	[46]
*A. hlangapies*	Isovitexin	[46]
*A. humilis*	Isovitexin, dihydroisorhamnetin	[46]
*A. inconspicua*	Isovitexin	[46]
*A. inyangensis*	Isovitexin	[46]
*A. kraussii*	Isovitexin	[46]
*A. linearifolia*	Isovitexin	[46]
*A. lineate*	Dihydroisorhamnetin	[46]
*A. macra*	Phenols, saponins, tannins, alkaloids, anthraquinones, terpenes, coumarins, and flavonoids in traces amount in comparison to A	[41]
*A. minima*	Isovitexin	[46]
*A. nubigena*	Isovitexin	[46]
*A. parviflora*	Isovitexin	[46]
*A. polyphylla*	Isovitexin and nataloin A and B	[46]
*A. pratensis*	Aloesin	[46]
*A. pretoriensis*	Isovitexin and dihydroisorhamnetin	[46]
*A. purpurea*	3-*O*-caffeoylquinic acid, aloesin, 4-*O*-*p*-coumaroylquinic acid, isoorientin pentoside, vitexin/isovitexin hexoside, vitexin/isovitexin pentoside, vitexin/isovitexin, aloin, 2″-*O*-trans-*p*-coumaroylaloenin, aloin B, aloeresin A, malonylnataloin, and aloeemodin dianthrone di-*O*-hexoside	[47]
*A. saundersiae*	Isovitexin	[46]
*A. soutpansbergensis*	Isovitexin	[46]
*A. striatula*	Aloeresin A and homonataloin A and B (chromones)	[46]
*A. suprafoliata*	Isovitexin, aloin A and B, and nataloin A and B	[46]
*A. suzannae*	Isovitexin, apigenin, and naringenin	[46]
*A. tenuior*	Isovitexin and homonataloin A and B	[46]
*A. thompsoniae*	Isovitexin	[46]
*A. thorncroftii*	Isovitexin and dihydroisorhamnetin	[46]
*A. tidmarshii*	Isovitexin	[46]
*A. vaotsanda*	Naringenin, dihydroisorhamnetin, aloesin, aloin A and B	[46]
*A. verecunda*	Isovitexin	[46]
*A. vossii*	Isovitexin	[46]

**Table 4:** Phytoconstituents of *Aloe* species extracted with different methods.

*Aloe* Species	Investigated Methods	Phytochemicals	Reference
**Leaves**			
*A. adigratana*	Solvent increasing polarity-gel extraction	Alkaloids, flavonoids, tannins, polyphenolic, glycosides, terpenoids, steroids, carbohydrates, amino acids, and proteins	[48]
*A. arborescens*	TLC pre-coated plates	Barbaloin, aloeresin, and aloenin	[28]
	Colorimetric assay, triple quadrupole and time-of-flight mass spectrometry, UPLC/Q-ToF high resolution mass spectrometry	Chromones (aloesin, aloesone, 8-C-glucosyl-noreugenin, aloeresin E, and 7-hydroxy-2,5-dimethylchromone); anthrones (aloin, aloe-barbendol, and aloesaponarin II); phenolic naphthalene (feroxidin); phenolic dimer (feralolide); flavonoids (naringenin, isovitexin, isorhamnetin, daidzenin, and genistein), and hydroxycinnamic acids (feruloylquinic acid, sinapic acid, chlorogenic acid, ferulic acid, and caffeic acid)	[49]
	Phytochemical screening	Flavonoids, terpeneoids, and aromatic compounds	[50]
*A. barbadensis*	Colorimetric assay	Glucose, galactose, mannose, and arabinose	
	GC-IT-MS; UPLC-Q-ToF-MS	Alanine, valine, succinic acid, arabitol, malic acid, pyroglutamic acid, aspartic acid, γ-aminobutyric acid, arabinose, fructose, glucose, glucuronic acid, sucrose, aloesin, homonataloside, 7-hydroxy-8-*O*-methylaloin, 7-hydroxyaloin B, 7-hydroxyaloin A, nataoemodin, aloeresin A, aloin B, isoaloeresin D, 7-*O*-methylaloeresin A, aloin A, 6′-malonylnataloin B, and 6′-malonylnataloin A	[51]
	Recrystallization, semi-preparative HPLC, or column chromatography	Chrysophanol, aloe-emodin, 7-hydroxy-2,5-dimethylchromone, 5-(hydroxymethyl)-7- methoxy-2-methyl chromone, saiko-chromone A, 5-((4E)-2′-oxopentenyl)-2-hydroxymethylchromone, 7-hydroxy-5-(hydroxymethyl)-2-methylchromone, aloenin aglycone, 5-((*S*)-2′-oxo-4′-hydroxypentyl)-2-hydroxymethylchromone, aloenin-2′-*p*-coumaroyl ester, 10-hydroxyaloin B, 10-hydroxyaloin A, isoaloeresin D, aloin B and A, aloesin, 8-C-glucosyl-I-aloesol, 8-*C*-glucosyl-7-*O*-methyl-(S)-aloesol, 10-*O*-β-d-glucopyranosyl aloenin, 5-((S)-2′-oxo-4′-hydroxypentyl)-2-(β-glucopyranosyl-oxy-methyl)-chromone, and aloenin B	[52]
	LCMS-IT-TOF; HPLC-DAD	Chromones (aloesin, 8-*C*-glucosyl-I-aloesol, 8-*C*-glucosyl-7-*O*-methyl-(S)-aloesol, isoaloeresin, 5-((S)-2′-oxo-4′-hydroxypentyl)-2-(β-glucopyranosyl-oxy-methyl) chromone, and 5-((S)-2′-oxo-4′-hydroxypentyl)-2-methoxychromone); phenyl pyrones (10-*O*-β-d-glucopyranosyl aloenin and aloenin-2′-*p*-coumaroyl ester); anthrones (aloin A and aloe-emodin), and naphthalene derivative (aloveroside B), aloesin, 8-*C*-glucosyl-I-aloesol, 8-*C*-glucosyl-7-*O*-(S)-methyl-aloesol, 10-*O*-β-d-clucopyranosyl aloenin, 5-((S)-2′-oxo-4′- hydroxypentyl-2(β-glucopyranosyl-oxy-methyl)chromone, 5-((S)-2′-oxo-4′-hydroxypentyl)-2-methoxy chromone, aloenin, 10-hydroxyaloin B, 10-hydroxyaloin A, aloveroside B, aloenin B, isoaloeresin D, aloin B, aloin A, aloenin-2′-p-coumaroyl ester, (*E*)-2-acetonyl-8-[(2″-*O*-cinnamoyl)-β-d-glucopyranosyl-7-methoxy-5-methylchromone, aloinoside B, aloinoside A, (*E*)-2-((S)-2-hydroxypropyl)-8-(2′-*O*-OCH_3_-cinnamoyl)-β-d-glucopyranosyl-7-methoxy-5-methyl-chromone, and aloe-emodin	[53]
	Phytochemical screening	Alkaloids, terpenoids, steroids, flavonoids, tannins, and reducing sugars	[54]
	Colorimetric assay, triple quadrupole and time-of-flight mass spectrometry, UPLC/Q-ToF high-resolution mass spectrometry	Chromones (aloesin, aloesone, 8-*C*-glucosyl-noreugenin, and aloeresin E); anthrones (aloin, aloe-barbendol, and aloesaponarin II); phenolic naphthalene (feroxidin); phenolic dimer (feralolide); flavonoids (isovitexin and isorhamnetin; isoflavones (daidzein and genistein); hydroxycinnamic acids (chlorogenic acid, ferulic acid, and caffeic acid)	[49]
*A. calidophila*	TLC, IR, MS, 1H NMR, and 13C NMR	Aloinoside, aloin, and microdontin	[55]
*A. ferox*	VLC fractionation, silica gel chromatography	Aloe emodin, chrysophanol, and aloin A	[56]
	Solvent partitioning and chromatography	Aloe-emodin, *p*-hydroxybenzaldehyde, *p*-hydroxyacetophenone, pyrocatechol, 10-oxooctadecanoic acid, 10-hydroxyoctadecanoic acid, methyl 10-hydroxyoctadecanoate, 7-hydroxy-2,5-dimethylchromone, furoaloesone, and 2-acetonyl-8-(2-furoylmethyl)-7-hydroxy-5-methylchromone	[57]
	Phytochemical screening	Flavonoids, condensed tannins, and gallotannins	[58]
	Phytochemical screening	Phenols, flavonoids, flavonols, proanthocyanidins, tannins, alkaloids, and saponins	[59]
	Fractionation, chromatography	Aloe emodin, aloin A, aloin B, desoxyaloin, aloinoside B, aloinoside C, aloinoside D, aloenin aglycone, feroxidin, 7-hydroxy-5-(hydroxymethyl)-2-methylchromone, 5-methylresorcinol, aloe resin D, 7-*O*-methylaloesinol, aloeresin G, *C*-2′-decoumaroylaloeresin G, 5-((*S*)-2′-oxo-4′-hydrosypentyl)-2-hydroxymethylchromone, aloveroside A, and aloenin B	[60]
*A. greatheadii* var. *davyana*	Solvent fractionation, GC-MS	Alkaloids (hypoxanthine), polyphenols/phenolic compounds (phenol, 4-ethylphenol, cholestenol, homovanilic, gentisic, 6,7-dihydroxycoumaric, o-hydroxycinnamic, protocatechuic, 3,4-dihydroxyphenylacetic, syringic, sinapic, caffeic, isoferulic, ferulic, benzoic, phenylacetic, 2-methoxybenzoic, *o*-toluic, phenylpropionic, 4-phenyllactic, 4-hydroxybenzoic, 2,3-dihydrobenzoic, 4-hydroxyphenylacetic, hydro-*p*-coumaric, and *p*-coumaric); phytosterols (cholestenol, campesterol, â-sitosterol, and stigmasterol)	[44]
*A. lomatophyllloides*	LC-UV-MS/MS	3-*O*-Caffeoylquinic acid, 4-*O*-*p*-coumaroylquinic acid, isoorientin pentoside, isoorientin, vitexin/isovitexin hexoside, vitexin/isovitexin pentoside, vitexin/isovitexin, aloin or nataloin isomer, aloin B, aloin A, aloeresin A, malonylnataloin, and aloeemodin dianthrone di-*O*-hexoside	[47]
*A. macra*	LC-UV-MS/MS	3-*O*-Caffeoylquinic acid, aloesin, 4-*O*-*p*-coumaroylquinic acid, vitexin/isovitexin hexoside, isoorientin pentoside, isoorientin, vitexin/isovitexin hexoside, vitexin/isovitexin pentoside, vitexin/isovitexin, aloin, 2″-*O*-trans-*p*-coumaroylaloenin, aloin B, aloin A, aloeresin A, and malonylnataloin	[47]
*A. marlothii*	FCC, TLC	7-*O*-methylaloeresin A, 5-hydroxyaloin A 6′-*O*-acetate, and 5-hydroxyaloin A	[61]
*A. rupestris*	FCC, TLC	7-*O*-methylaloesin and aloesin	[61]
*A. sabaea*	Phytochemical screening	Glycoprotein	[62]
*A. striata*	Phytochemical screening	Flavonoids, terpeneoids, and aromatic compounds	[50]
*A. tormentorii*	LC-UV-MS/MS	Aloesin, 4-*O*-*p*-coumaroylquinic acid, vitexin/isovitexin hexoside, isoorientin pentoside, isoorientin, vitexin/isovitexin hexoside, vitexin/isovitexin pentoside, vitexin/isovitexin, aloin or nataloin isomer, 2-*O*-*trans*-*p*-coumaroylaloenin, aloin B, aloin A, aloeresin A, malonylnataloin, aloeemodin dianthrone di-*O*-hexoside, and microdontin A or B	[47]
*A. trichosantha*	TLC	Aloin A/B and aloin-6′-*O*-acetate A/B	[63]
*A. vera*	Phytochemical screening	Steroids, terpenoids, carotenoids, anthraquinones, catechin, and tannins	[64]
	Phytochemical analysis	Alkaloids, glycosides, reducing sugars, phenolic compounds, steroids, terpenoids, flavonoids, tannins, and saponin glycosides	[65]
	LC-UV-MS/MS	3-*O*-Caffeoylquinic acid, aloesin, 4-*O*-*p*-coumaroylquinic acid, vitexin/isovitexin hexoside, isoorientin, vitexin/isovitexin hexoside, vitexin/isovitexin pentoside, vitexin/isovitexin, aloin, 2″-*O*-feruloylaloesin, 7-*O*-methylaloeresin A, aloin B, aloin A, malonylnataloin, and microdontin	[47]
	Chromatography	Aloeresin G, isoaloeresin D, aloeemodin, babarloin A, 8-*O*-methyl-7-hydroxyaloin B, elgonica-dimer A, elgonica-dimer B, feralolide, hopan-3-ol, β-sitosterol, and daucosterol	[66]
	Solvent fractionation, TLC, GC-MS	Pyrocatechol, cinnamic acid, *p*-coumaric acid, and ascorbic acid	[67]
**Roots**			
*A. pulcherrima*	Fractionation, column chromatography	Chrysophanol, aloesaponarin II, and aloesaponarin I	[68]
*A. megalacantha*	Column chromatography	Chrysophanol, helminthosporin, and methyl 26-*O*-feruloyl-oxyhexacosanoate; asphodelin, aloesaponarin III, and 10-(chrysophanol-70-yl)-10-hydroxychrysophanol-9-anthrone; aloesaponarin I, aloesaponarin II, 4,7-dihydroxy-5-methylcoumarin, 1,8-dimethoxynepodinol, and aloe emodin; 10-*O*-methylchrysalodin and chrysalodin, aloesaponol	[69]
**Whole Plant**			
*A. turkanensis*	Phytochemical screening	Tannins, anthraquinones, terpenoids/steroids, saponins, and alkaloids	[70]
*A. barberae*	TLC	Aloin and chrysophanol	[71]
**Leaves and roots**			
*A. arborescens* var. *natalensis*	Silica gel chromatography, TLC, GC-MS	Aloe-emodin, barbaloin, 2″,-*O*-feruloylaloesin, aloenin, aloesin, succinic acid, d-glucose, fatty acid methyl esters, *n*-triacontanol, *n*-dotriacontanol, and â-sitosterol	[72]

UPLC/Q-Tof: ultra-performance liquid chromatography quadrupole time-of-fligh; IT: ion trap; DAD: diode-array detection; VLC: vacuum liquid chromatography; LC-UV-MS/MS: liquid chromatography with ultraviolet detection and tandem mass spectrometry; FCC: flash column chromatography.

**Table 5:** Phytoconstituents of *Aloe* species extracted with non-specified methods.

*Aloe* Species	Phytochemicals	Reference
**Leaves**		
*A. aageodonta*	7-*O*-Methylaloesin, aloeresin D, aloin A, aloin B, aloinoside A, aloinoside B, microdontin A, and microdontin B	[33]
*A. barbadensis*	Cycloartenol, 24-methylene-cycloartanol, lophenol, 24-methyl-lophenol, and 24-ethyl-lophenol	[73,74,75]
	Anthraglycosides, sugars, cardiotonic glycosides, mucilages, pectin, sterols type Δ^5^, anthraquinones, saponins, sterols, and triterpenoids	[76]
*A. castanea*	6′-*O*-Coumaroylaloesin	[77]
*A. claviflora*	Oxanthrone, 10-hydroxyaloin B 6′-*O*-acetate	[78]
*A. excelsa*	Aloe-emodin and aloin A	[79]
	Aloctin A and aloctin B	[80]
	1,8-Dihydroxy-3-methylanthracenedione (chrysophanol),	[79]
*A. ferox*	Aloeresin D (a C-glucosylated 5-methylchromone), feroxidin (1-methyltetralin derivative), and feralolide (a dihydroisocoumarin)	[81,82,83]
	5-Hydroxy-3-methylnaphtho[2,3-c]furan-4(9*H*)-one, 5-hydroxy-3-methylnaphtho[2,3-c]furan-4,9-dione, and 5-hydroxy-3-methylnaphtho[2,3-c]furan-4(1*H*)-one	[84]
*A. microstigma*	5-Hydroxyaloin A and microstigmin A,	[85]
*A. nyeriensis*	Aloesin, 7-*O*-methylaloesin, aloenin, aloeresin D, aloin B, and aloin A	[34]
*A. purpurea*	Phenols and trace amounts of saponins, tannins, alkaloids, anthraquinones, terpenes, coumarins, and flavonoids	[41]
*A. rabaiensis*	Aloe-emodin-11-*O*-rhamnoside, aloe-emodin anthrone-10-*C*-glucoside, aloe-emodin anthrone-10-*C*-rhamnoside, aloeresin D, and rabaichromone	[86]
*A. rubroviolacea*	Phytosterols (cholesterol, 24-methylcholesta-5,22-dien-3β-ol, campesterol, campestanol, stigmasterol, 15holesteno, and sitostanol); anthraquinones (aloin A and aloe-emodin); anthrone-anthraquinones (elgonica A and elgonica B); *C*-glycosyl chromones (8-*C*-glycosyl-(2’-*O*-cinnamoyl)-7-*O*-methylaloediol B, aloeresin E, and 8-*C*-glycosyl-7-*O*-methyl-I-aloesol)	[87]
*A. sabaea*	Coniine, γ-coniceine, N-4′-chlorobutylbutyramide, and *N*,*N*-dimethylconiine	[88]
*A. vera*	Tannins, saponins, and flavonoids	[89]
	β-sitosterol	[25]
**Roots**		
*A. berhana*	Aloesaponol I, laccaic acid D methyl ester, aloesaponol III, aloesaponarin I, chrysophanol-8-methyl ether, chrysophanol, and aloechrysone	[90]
**Stems**		
*A. saponaria*	Aloesaponarin I, aloesaponarin II, desoxyerythrolaccin, helminthosporin, isoxanthorin, and laccaic acid D methyl ester, aloesaponol III, aloesaponol IV, chrysophanol, helminthosporin, and isoxanthorin	[91]
**Flowers**		
*A. perryi*	Glycosides, phytosterols, proteins, and amino acids, flavonoids, phenols, and carbohydrates	[92]
**Leaves and roots**		
*A. hijazensis*	Aloe-emodin, emodin, chrysophanol, aloesaponarin II, 3-methyl ether, ziganein, ziganein-5-methyl ether, aloesaponarin I, chrysophanein, feralolide, 4,7-dichloro-quinoline, lupeol, aloin, aloenin, ethylidene-aloenin, aloenin B, quercetin, kaempferol, cosmosiin, isovitexin, cinnamic acid, caffeic acid, and ferulic acid	[93]
*A. arborescens* var. *natalensis*	2′-*O*-*p*-Coumaroylaloesin and 2′-*O*-feruloylaloesin	[94]

**Table 6:** Bioactive effects of *Aloe* plant species: pre-clinical (in vitro and in vivo) studies.

Biological Activities	Observed Effects	Active Molecules	References
Wound healing and cell proliferation	Skin damage treatment, cell type proliferation stimulation, cell phagocytic activity stimulation, wound contraction rate, collagen and elastin synthesis increase, fibroblast proliferation, hyaluronic acid, and hydroxyproline production	Mannose-6-phosphate, polysaccharides, glycoproteins, saponins, acemannan	[5,33,135,174,175,176,178,179,180,184,185,186]
Intestinal absorption and purgative action	Drug permeability increase (opening tight junctions), intestinal water absorption reduction, mucus secretion stimulation, reduce visceral fat accumulation, reduce large-sized intestinal polyps, intestinal motility improvement	Anthraquinones (aloin, aloe-emodin, emodin), pytosterols	[5,133,188,189,190,191,192,194]
Anti-inflammatory and immunomodulatory	Phagocytic and prolifertive activity raise (through cyclooxygenase (COX) pathways inhibition and prostaglandins production reduction), abolish albumin gene transcription, inflammatory processes inhibition (leukocyte adhesion and pro-inflammatory cytokines production reduction), cerebral ischemia and reperfusion injury attenuation (inhibition of systemic inflammatory response, leukocyte aggregation, and lipid peroxidation)	Aloe-emodin, polysaccharides, aloesin, anthraquinones, chromones	[2,148,199,200,201,202,203,204,205,206,207,208]
Hepatoprotective	Morphofunctional and molecular changes reduction, protection against hepatocyte death and lipid peroxidation, liver fatty acid synthesis downregulation and oxidation upregulation, cytokine level reduction, mRNA lipogenic gene expression suppression	Aloe-emodin, anthraquinones, phytosterols (lophenol, cycloartanol)	[74,133,209]
Antioxidant	Free radical scavenging activity, free radicals’ generation and reactive oxygen species (ROS) production suppression, lipid peroxidation reduction, superoxide dismutase (SOD) activity raise	Aloesin, aloeresin A, and aloesone	[44,49,198,212,213,214,215,216,217,218]
Antibacterial, antifungal and antiviral	Phagocytic leukocyte activity stimulation, cytotoxic effects, alkalization promotion and constipation alleviation, virus replication inhibition	Emodin, aloin A, aloe-emodin, saponins, chrysophanol, acemannan, pyrocatechol, polysaccharides	[6,56,58,67,71,76,79,136,146,148,170,181,192,198,219,220,221,222,223,224,225,226,227,228,229,230,231,232,233]
Anticancer	Chemopreventive activity, VEGF secretion inhibition, tumor angiogenesis and angiogenic response inhibition, proliferation inhibition and endothelial cell migration, N-acetyl transferase activity and gene expression inhibition, STAT3 activation blocking, benzopyrene binding, iNOS, NFkB, and P53 activity inhibition, TNF-α, IL-1, and interferon production stimulation	Aloin, aloe-emodin, rhein, acemannan, barbaloin, physcion, chrysophanol, aloesin, diethyl hexylphthalate and an N-terminal octapeptide	[148,184,225,234,235,236,237,238,239,240,241,242,243,244,245,246,247,248,249,250,251,252,253,254,255]
Antidiabetic	Glucose transporter mRNA expression modulation, reduce fasting blood glucose levels, glucose transport improvement through proximal and distal marker modulation	Polysaccharides, phytosterols (lophenol, 24-methyl-lophenol, 24-ethyl-lophenol, cycloartanol and 24-methylene cycloartenol), aloe-emodin-8-*O*-glycoside	[37,73,74,148,183,255,256,257,258,259,260]
Antihyperlipidemic	Reduce visceral fat mass, total cholesterol, triglycerides, LDL and VLDL levels, glucose intolerance and lipid metabolizing enzymes improvement and abnormal estrous cyclicity reversion	Phytosterols	[261,262,263,264,265]
Estrogen status	Suppress breast cancer cells proliferation, estrogen receptor-α inhibition	Emodin, aloe-emodin	[52,263]
Antiulcer	Promote digestion, cytoprotection, dose-dependent gastric acid secretion inhibition	Plant extract	[5,266,267,268,269]
Skin use	Increase type I and type III collagen synthesis gene expression and hyaluronic acid levels, tyrosinase inhibitory activity,	Sterols, aloin, aloesin	[270,271,272,273,274,275]
Antiallergic	Reduce histamine release, stimulate leukotriene synthesis and secretion, protein kinase C and phospholipase C activities inhibition, Ca^2+^ influx blocking during mast cell activation	Glycoprotein	[276]

VEGF: vascular endothelial growth factor; iNOS: inducible nitric oxide synthase; NFκB: nuclear factor κB; TNF-α: tumor necrosis factor α; IL-1: interleukin 1; LDL: low density lipoprotein; VLDL: very low density lipoprotein.

**Table 7:** Bioactive effects of *Aloe* plant species: clinical studies.

Biological Activities	Observed Effects	Active Molecules	References
Wound healing and cell proliferation	Tyrosinase activity inhibition, re-epithelialization, wound healing promotion	Arbutin, aloesin	[310,311,312,313,314,315,316,317]
Anti-inflammatory and immunomodulatory effects	Decrease pain score and aphthous wound healing period, promote eye external part treatment, downregulate lipopolysaccharide-induced inflammatory cytokine production and NLRP3 inflammasome expression, potentiate lymphocyte response, phagocytosis and circulating monocyte and macrophage levels	Acemannan, polysaccharides	[75,254,266,318,319,320,321]
Antidiabetic effects	Lower blood glucose levels, reduce body weight, body fat mass and insulin resistance, revert impaired fasting glucose levels and impaired glucose tolerance	Plant extracts	[322,323]
Antihyperlipidemic effects	Reduce atherosclerosis, total serum cholesterol and LDL levels	Plant extracts	[323]
Acquired immune deficiency syndrome (AIDS) treatment	Sooth wound and burn of internal organs, inhibit HIV-1 virus	Mannose-6-phosphate, plant extract	[324]
Dental and oral diseases treatment	Heal aphthous ulcers and reduce pain, plaque and gingivitis	Acemannan	[325,326]
